# The growing burden of workplace violence against healthcare workers: trends in prevalence, risk factors, consequences, and prevention – a narrative review

**DOI:** 10.1016/j.eclinm.2024.102641

**Published:** 2024-05-27

**Authors:** Conor J. O'Brien, André A.J. van Zundert, Paul R. Barach

**Affiliations:** a The Department of Anaesthesia and Perioperative Medicine, Royal Brisbane and Women's Hospital, Herston Campus, Brisbane, QLD, Australia; b The University of Queensland, Faculty of Medicine, Herston, QLD 4006, Australia; c Thomas Jefferson University, Philadelphia, PA, United States

**Keywords:** Workplace violence, Healthcare workers, Aggression, Violence, Staff turnover, Prevention

## Abstract

Workplace violence (WPV) against healthcare workers (HCW) is a globally growing problem in healthcare systems. Despite decades of research and interventions violent incidents are rising in their severity and frequency.

A structured review of PubMed and Scopus databases and supplementary internet searches, resulted in a synthesis of evidence covering multiple countries and healthcare worker populations. High rates of WPV are increasingly common due to unmet patient expectations, poor communication, long wait times and organizational factors such as resourcing and infrastructure.

We highlight links between WPV and poor worker health outcomes, staff turnover, reduced patient safety and medical errors. Few prevention and mitigation activities have shown sustained effects, highlighting the challenges in understanding and addressing the complex interplay of factors that drive violence against HCWs.

The rapidly rising incidence of WPV requires special consideration and action from multiple stakeholders including patients and visitors, healthcare providers, law enforcement, media and policy makers.

## Introduction

Workplace violence (WPV) against healthcare workers (HCWs) is a global problem with a complex etiology. WPV has been documented in healthcare systems globally without a clear link to wealth, type of organization or cultural factors. Many healthcare organizations and clinicians have observed increasing rates of violence over recent years with a concomitant rise in negative impacts on HCW.^1^^,^^2^ Historically, mitigation efforts have failed to curb WPV leading to greater attention by media and policy makers with legislative action now a focal point for intervention.^3^ The spectrum of violent incidents range from verbal and emotional violence to physical injury and even death. Tragically, the literature describes recent incidents resulting in the death of more than 370 HCWs (Panel 1), with the true number undoubtedly much higher.^4^, ^5^, ^6^, ^7^, ^8^

*Panel 1* **Healthcare worker deaths.**

**The dire reality of HCW deaths**

• 156 healthcare workers were killed at their health workplaces between 2011 and 2018 in the U.S., averaging about 20 deaths each year.^4^
• Since 2017, at least ten doctors have been violently killed in the Philippines with doctors being subject to bounties and targeted because of perceived political affiliations.^5^
• Between 1988 and 2019, 21 doctors were killed in Italy by patients or their relatives.^6^
• In 2021, 161 medics were killed in 49 conflict zones.^7^
• In China, 101 incidents of serious medical violence occurred between 2003 and 2013, in which 24 doctors and nurses died.^8^

The genesis of this narrative review lies in the growing breadth, acuity and complexity of WPV which appears refractory to mitigation efforts. We attempt to provide insight into both historical and contemporary evidence of the multiple forces driving WPV as well as the impacts on HCWs and the healthcare system more broadly. This is achieved through synthesis of evidence across a variety of jurisdictions and healthcare contexts to identify trends and themes that may prove useful in the development and implementation of future interventions.

## Methods

### Overview

This narrative review included five key phases: identifying the research question, identifying relevant studies, study selection, collating data and synthesizing results. We attempted to follow the Preferred Reporting Items for Systematic Reviews and Meta–Analyses adjusted extension for scoping reviews (PRISMA–ScR) with amendments reflecting the characteristics of narrative reviews. Ethical approval was not required for this study.

The authors assessed the feasibility of undertaking a statistical analysis based on the method outlined in the Cochrane Handbook.^9^ This included quantitative assessment of risk of bias, weighting and stratification of study results, ratio or mean difference effect measures, calculating summary statistics and utilizing forest plots. Based on the qualitative nature of most included studies, the multiple outcomes assessed and the heterogeneity of methods and data it was deemed infeasible to undertake a statistical analysis.

### Study objectives

The objective of this narrative review is to map the prevalence, impacts and costs associated with WPV, assess the success of interventions, and provide recommendations to guide prevention and mitigation strategies. The primary aims are to determine trends across the outcomes of incidence, prevalence and causation in different HCW groups (primarily doctors and nurses) in hospitals and primary care settings and various cultural, economic and geographical contexts. The secondary aims are to better understand the impacts of WPV on the physical and psychological health of HCWs, access to healthcare services through economic and capacity constraints and nature and success of countermeasures.

### Search strategy and selection criteria

We searched PubMed (including MEDLINE) and Scopus databases in February 2023 with supplementary searches of these databases and Google occurring up to November 2023. The databases were selected to be comprehensive and cover a broad range of healthcare contexts. We included only papers published in English with no limits on publication dates, subject or study type. Table 1 provides the search query terms tailored to the specific requirements of each database.

**Table 1:** Databases and search terms.

Search date	Database	Search terms	Search function	Items returned
2/2/2023	PubMed	Workplace violence, healthcare, costs	((workplace violence) AND (healthcare)) AND (costs)	30
2/2/2023	PubMed	Workplace violence, healthcare, costs	((workplace violence) AND (healthcare) OR (health care)) AND (costs)	44
7/2/2023	PubMed	Workplace violence, healthcare workers, impacts	(workplace violence against healthcare worker) AND (impacts)	88
8/2/2023	Scopus	Workplace violence, healthcare workers	(workplace AND violence AND against AND healthcare AND workers)	182
13/2/2023	PubMed	Workplace violence, healthcare workers, intervention	((workplace violence) AND (healthcare worker)) AND (intervention)	944
13/2/2023	Scopus	Workplace violence, aggression, healthcare workers, interventions	workplace AND violence OR aggression AND healthcare AND workers AND interventions	117
13/2/2023	Scopus	Workplace violence, aggression, healthcare workers, impacts	workplace AND violence OR aggression AND healthcare AND workers AND impacts	96
13/2/2023	Scopus	Workplace violence, aggression, healthcare workers, cost	workplace AND violence OR aggression AND healthcare AND workers AND cost	27
17/2/2023	PubMed	Workplace violence, aggression, healthcare worker, doctor, nurse	(((workplace)) AND (violence OR aggression)) AND (healthcare worker OR doctor OR nurse)	2994
**Total**				**4522**

The initial selection for inclusion was based on a single reviewer assessment of titles and abstracts (CO). Both quantitative and qualitative items were eligible if they included prevalence of WPV against HCWs as the primary outcome or assessed causation, impacts or interventions. Articles were included based on their relevance to provide contemporary and comprehensive insights into the state of WPV, and the range and efficacy of interventions.

A full–text copy of each eligible study was examined. The authors used their experience to determine final study inclusion based on the objective of providing a comprehensive overview of WPV against HCW across multiple countries, healthcare systems and populations. The references of eligible studies were manually checked (snowballing), to identify additional relevant studies that were missed in the database searches. All selections and ongoing searches were closely reviewed by three authors (CO, AVZ and PB) with full agreement achieved before proceeding.

### Supplemental searches

Additional searches were undertaken in other sources of information such as media reports, guidelines, correspondence and opinion articles to provide contextual detail. This assisted in better understanding public and political attitudes toward WPV.

### Data extraction

Each article that met the study eligibility criteria was abstracted using a standardized form that tabulated publication date, study type, primary outcomes, incidence and prevalence, causes, risk factors, setting, perpetrator characteristics, impact on staff and healthcare system and prevention and mitigation strategies (see Supplementary Material Table S1).

### Role of the funding source

No funding was received for this study.

## Results

### Search results

**Fig. 1:**
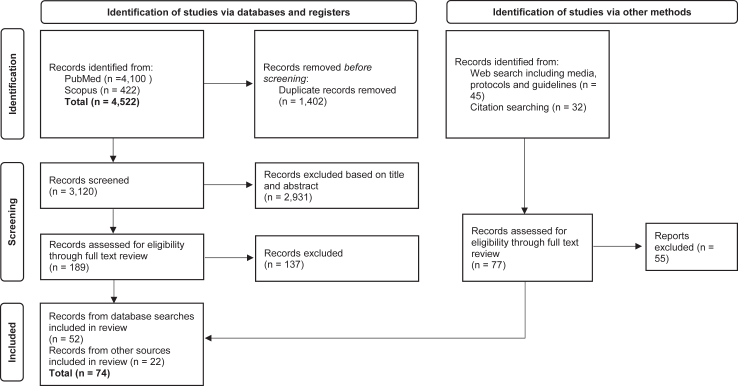
**Study search flow diagram**.

We identified 3197 citations (once duplicates were removed), of which 266 underwent full–text abstraction and further analysis, with 74 items ultimately incorporated into the review (Fig. 1). Because of heterogeneity of the study designs, participants and outcome measures, a meta–analysis was not feasible.

### Characteristics of included studies

A majority of the included studies involved the use of surveys and self–reporting to assess outcomes. We found significant heterogeneity among included studies with a variety of methodological differences in design and study populations. Although this limited the ability to make robust comparisons of outcomes it did afford the opportunity to identify broad trends in causes, risk factors, incidence and prevalence.

Not all included studies provide an explicit definition of WPV, however there are a number of common elements that can be used to form a working definition. These elements are,

1. Violence may include verbal threats or intimidation, physical assault or inappropriate conduct of an emotional or sexual nature.
2. The action is either intentional, in the case of a perpetrator with cognitive capacity, or the result of pathological processes or intoxication, in the case of an individual without capacity.
3. The action has, or is likely to have, a negative impact on the safety and physical, mental or emotional wellbeing of the HCW. And
4. The action occurs in the context of the HCW performing their duties or where the victim can be identified as a HCW.

These elements capture the breadth of behaviors and impacts studied in the literature and provide insight into the extent and complexity of WPV.

### Quality assessment of methods

A high level assessment of methodological quality was undertaken using critical appraisal tools and methodologies published by the Joanna Briggs Institute (JBI),^10^, ^11^, ^12^ the Cochrane Qualitative Evidence Synthesis protocol^13^ and GRADE-CERQual^14^ with adjustments to reflect a narrative review standard. A Qualitative Evidence Profile for the major outcomes described in this study is provided in Table 2.

**Table 2:** Qualitative evidence profile.

Narrative review finding	Methodological limitations	Coherence of findings	Adequacy of data	Relevance of data	Assessment of confidence	Explanation of assessment
Incidence/prevalence of workplace violence against healthcare workers has increased with evidence physical violence has a smaller proportion of incidence/prevalence however may have been increasing at a high rate.	Moderate to major concerns regarding risk of bias, particularly in relation to convenience sampling, retrospectivity of exposure and lack of verification of subject responses. Lack of appropriate statistical analysis of quantitative data.	Minor concerns regarding coherence with a majority of studies having findings rationally derived from included data.	Minor to moderate concerns regarding adequacy with a range of study sample and population sizes with variability in the number of outcome measures ranging from single dimensional definitions of WPV to multi-dimensional definitions with a number of different data points for types of WPV.	Minor concerns regarding relevance with most studies providing contemporary data related to the key elements of WPV definition.	Moderate confidence.	There is a wide range of studies covering multiple populations and jurisdictions and consistency with the key elements of WPV definitions. The primary concern relates to reliability of data due to the biases inherent in retrospective self reporting of cross-sectional studies with convenience sampling.
There are multiple dimensions of causation of WPV including patient/visitor, HCW, organizational/socio-cultural with co-dependences between these dimensions. The factors which play a significant role in WPV include poor communication, resource shortages, patient expectations, wait times, and commercialization of healthcare.	Moderate to major concerns. Cross-sectional and systematic reviews relying on inherent biases in data collection methods and text and opinion articles with limited methodological quality	Moderate to major concerns with speculative nature of victims reports of causation and inability to reliably verify causation from perpetrator's perspective.	Major concerns with lack of majority of studies providing qualitative inference based on victim only perspectives of causation.	Minor concerns with most studies reporting causation within a small range of themes factors.	Low confidence.	No studies were able to provide empirical evidence of the causative factors driving reported WPV. Outcomes are based on the views and experiences of victims with little attention given to the perspective of perpetrators.
There are mental and physical health impacts of workplace violence against healthcare workers leading to impacts on quality and access to care through HCW performance and reduced HCW resources.	Moderate concerns. Despite being supported by robust statistical analysis in some studies the limitations of self reporting and lack formal diagnostic processes for reported conditions limits evidence quality. Similar concerns exist for staff performance with self reporting errors and risks.	Minor concerns with the study findings reflecting the reported data.	Moderate concerns. Relatively few studies contributed to this outcome with primary data although a significant number mention it as a theme or issue in discussion.	Minor concerns with studies coming from a range of contexts and populations.	Low confidence.	The lack of formal diagnostic evidence of reported mental and physical health impacts severely limits the reliability of this finding. However it is reasonable to assert that WPV has such impacts based on subjective reporting given the nature of the mental health conditions and known associations with physical symptoms.
There is a significant cost of workplace violence against healthcare workers in relation to loss of staff which includes direct costs associated with re-hiring, as well as further exacerbating risks associated with limited health resources.	Minor concerns. Few studies report costs associated with staff losses however they are relatively reliable. Less confidence associated with self reported attrition and intention to leave.	Minor concerns. Findings generally well supported by included data.	Moderate concerns. Relatively few studies contributed to this outcome and primarily focused on China. However, data and forecasting from credible global organizations such as the WHO support the critical impact staff shortages will have on access to healthcare.	Minor concerns. The data and findings are supported by a common narrative from global health organizations around risks associated with availability of resources and access to healthcare.	Moderate confidence.	A relatively small number of included studies however the data and outcomes are reasonably well supported by methodological considerations and are consistent with reporting from credible global health organizations.
Current interventions have not been effective at reducing rates of WPV.	Moderate to minor concerns. Includes a number of randomized and non-randomized trials, some high quality systematic reviews and reports and guidelines by credible organizations. Underlying data is reasonably robust and in some instances based on standardized organizational reporting data, however there is still an element of bias with retrospective self-reporting.	Minor concerns. Findings generally well supported by included data and demonstrate consistency across interventions targeted at different stakeholders and activities.	Minor concerns. There is significant data obtained through both well established formal reporting mechanisms and more subjective self reporting mechanisms.	Minor concerns. Multiple jurisdictions assessed and various interventions through a range of different study methodologies.	Moderate to high confidence.	There are a wide range of studies across multiple jurisdictions targeting different intervention types and stakeholders. This is further supported by narratives and guidelines put forward by credible global health organizations.
Underreporting of WPV is a significant and persistent problem and it is unclear to what extent interventions have increased the reported rates of WPV.	Moderate concerns. Few studies reporting underreporting data however it is a consistent narrative and supported by relatively robust studies that suffer less from methodological deficiencies.	Minor concerns. Findings generally well supported by data however some difficulties defining the true extent of the issue.	Moderate concerns. Although a common theme, a proportion of the data is narrative in nature.	Minor concerns. A common issue described almost ubiquitously across health systems.	Moderate to high confidence.	Although some studies provided empirical data this is a common outcome described in study narratives and is supported by multiple credible stakeholders and organizations across many decades.

This assessment was undertaken by a single reviewer (CO) to identify general trends or limitations in the quality of existing literature. The quality of evidence is considered low to moderate confidence across the outcome domains of incidence and prevalence, causation, and mental and physical health impacts, owing primarily to the self-reporting, retrospective nature of much of the data collection. This is particularly evident for mental and physical health impacts which suffer from lack of formal diagnostic processes. Additionally, few studies discuss, or attempt to mitigate, confounding factors and bias making a causal relationship between WPV, antecedent factors and consequences difficult to establish.

Moderate confidence is ascribed to outcomes associated with impacts of WPV, intervention efficacy and underreporting. These outcomes are better supported by included study methodologies and consistency in qualitative evidence across multiple jurisdictions and healthcare contexts.

### Causes and risks for workplace violence (WPV) against healthcare workers (HCWs)

The nature of workplace violence and the methods used to study it makes identifying causation difficult. The approach of this review is to identify trends and themes across different healthcare contexts to give organizations and researchers a framework for assessment and investigation of their unique workplace violence problem. A myriad of causes, risk factors and risk markers for WPV are described in the literature. Tables 3 and 4 provide tabulated data on these elements and provide insights into the major themes identified in the literature. The review identified three main dimensions that were reported frequently in included studies and provide an early framework to understand the drivers of WPV. These dimensions are patient/visitor, HCWs, and organizational/socio–cultural. Significant co–dependence was observed between the dimensions, and we noted that local contextual factors can impact their relative contributions to WPV in a particular setting.

**Table 3:** Synthesis of evidence for causes of workplace violence against healthcare workers.

Study, year (reference)	Patient/visitor								HCWs		Organizational/socio-cultural			
	Unmet expectations	Dissatisfaction with treatment	Unmet demands	Stress	Acute illness	Intoxication	Psychiatric illness	Delirium/dementia	Poor communication	Discomfort associated with care	Long wait times	Insufficient staffing and resources	Healthcare costs	Restrictions (visitation/movement)
Kumar et al., 2016^15^											X		X	
Duan et al., 2019^16^												X		
Nowrouzi-Kia et al., 2019^17^	X					X	X		X		X	X		
Byon et al., 2021^18^			X									X		
Aljohani et al., 2021^19^						X	X				X	X		
Civilotti et al., 2021^20^	X	X				X	X	X	X	X	X			
Spelten et al., 2022^21^	X										X	X		
Ramzi et al., 2022^22^			X	X	X							X		
Lei et al., 2022^23^											X	X	X	
El-Zoghby et al., 2022^24^	X		X								X			
Vento et al., 2020^25^	X	X							X		X	X		
Caruso et al., 2022^26^									X			X	X	X
Iacobucci, 2022^27^	X										X			
Dopelt et al., 2022^28^						X			X	X	X	X		X
Viottini et al., 2020^29^	X					X	X	X	X

**Table 4:** Synthesis of evidence of risk factors and risk markers for workplace violence against healthcare workers.

Study, year (reference)	Patient/visitor		HCWs											Organizational/socio-cultural			
	Uncertain care	Less educated	Emergency department	Mental health facility	Evening/night shifts	Increased patient contact	Male	Female	Heavy workload	Younger age, less experience	Lack of training	Remote areas	Poor organizational support	Inadequate security	Media/political influence	Lack of resources	Higher costs
Kumar et al., 2016^15^					X					X							
Duan et al., 2019^16^																X	
Nowrouzi-Kia et al., 2019^17^											X	X	X	X		X	
Byon et al., 2021^18^																X	
Aljohani et al., 2021^19^							X			X			X			X	
Civilotti et al., 2021^20^								X									
Spelten et al., 2022^21^			X													X	
Ramzi et al., 2022^22^	X																
Lei et al., 2022^23^		X	X		X	X	X										
El-Zoghby et al., 2022^24^			X		X				X				X	X	X		
Vento et al., 2020^25^															X		
Caruso et al., 2022^26^								X	X	X	X		X				X
Iacobucci, 2022^27^															X	X	
Dopelt et al., 2022^28^																X	
Viottini et al., 2020^29^					X					X							
Liu et al., 2019^30^			X	X					X	X							
Tian et al., 2021^31^							X										
Tiesman et al., 2022^32^															X		

A majority of studies identify organizational factors as playing a major role in WPV, in particular insufficient staffing and resources to meet patient demands, long patient wait times, lack of appropriate security and poor organizational support for staff.^15^, ^16^, ^17^, ^18^, ^19^, ^20^, ^21^, ^22^, ^23^, ^24^, ^25^, ^26^, ^27^, ^28^, ^29^ Intoxication and cognitive changes associated with disease are commonly reported.^17^^,^^19^^,^^20^^,^^28^^,^^29^ HCWs working in emergency departments, night shifts and those of younger age and less experience appear to be at a much higher risk of injury.^15^^,^^21^^,^^23^^,^^24^^,^^29^^,^^30^

Emerging social and cultural factors have changed the nature of the relationships between the public, healthcare system and HCWs. Patient's expectations of access, expediency and efficacy of care appears to have increased, as health information has proliferated.^17^^,^^20^^,^^21^^,^^24^^,^^25^^,^^27^^,^^29^ Expectations and attitudes are being shaped, both positively and negatively, by political stakeholders who see strategic value in either promoting or undermining health services for political gain.^26^ This is exacerbated by media reporting that seeks to sensationalize rare events giving false perceptions of increased risks of medical errors and capacity constraints.^27^ These, along with other factors including increasing costs of care and commercialization^15^^,^^23^^,^^26^ are changing the attitudes of patients and visitors which may be contributing to the prevalence of WPV.

Poor communication between clinicians and patients also features prominently as a cause of WPV.^17^^,^^20^^,^^25^^,^^26^^,^^28^^,^^29^ There are reported associations between effective communication, age, experience and levels of training that make young, inexperienced HCWs more susceptible to WPV. Other demographic factors are reported as risk markers including females being at greater risk of non-physical violence and sexual harassment while males are at higher risk of physical violence.^19^^,^^20^^,^^23^, ^24^, ^25^^,^^31^

### Prevalence of workplace violence (WPV) against healthcare workers (HCWs)

Included studies report a wide range in prevalence of WPV against HCWs, owing to significant population and methodological differences. Although data synthesis was complicated by heterogeneity and subjective reporting, concerning trends emerge from the literature with a number of studies reporting a 12-month prevalence of non–physical violence above 90%.^20^^,^^21^^,^^26^^,^^33^ Table 5 provides the prevalence of commonly reported types of WPV in the reviewed literature.

**Table 5:** Prevalence of various types of workplace violence against healthcare workers.

Study, year (reference)	Study type	Location	Any type (%)	Verbal (%)	Physical (%)	Sexual harassment (%)	Type
Kumar et al., 2016^15^	CSS	India	–	87.3	8.5	–	12-month prevalence
Duan et al., 2019^16^	CSS	China	66.2	65.3	12.6	0.9	12-month prevalence
Nowrouzi-Kia et al., 2019^17^	SR-MA	Multi-country	69	–	–	–	Prevalence from 6 study meta-analysis
Byon et al., 2021^18^	CSS	United States	–	67.8	44.4	–	5-month prevalence during COVID-19
							pandemic (Feb–June 2020)
Aljohani et al., 2021^19^	SR-MA	Multi-country	–	72	18	9.5	Prevalence from meta-analysis of 5792 HCWs
Civilotti et al., 2021^20^	Scop	Italy	–	11.9–93.3	0–53	–	12-month prevalence
Civilotti et al., 2021^20^	Scop	Italy	–	48.8–90.9	25.7–64.7	–	Career prevalence
Spelten et al., 2022^21^	CSS	Canada	95	–	–	–	Estimated prevalence
Ramzi et al., 2022^22^	SR-MA	Asia, Americas Europe	47	44	17	–	Prevalence meta-analysis of 17,207 HCWs
Lei et al., 2022^23^	CSS	China	79.4	78.4	39.7	–	12-month prevalence
El-Zoghby et al., 2022^24^	CSS	Egypt	84	–	–	–	Prevalence
Vento et al., 2020^25^	Op	Multi-country	–	57.6	–	12.4	Prevalence
Caruso et al., 2022^26^	NR	Multi-country	70	–	–	–	12-month prevalence
Caruso et al., 2022^26^	NR	Multi-country	75–90	–	–	–	Career prevalence
Dopelt et al., 2022^28^	CSS	Israel	71	69	11	–	6-month prevalence
Viottini et al., 2020^29^	OS	Italy	3.3	–	–	–	Reported through hospital reporting system
Liu et al., 2019^30^	SR-MA	Multi-country	61.9	42.5	24.4	–	12-month prevalence
Tian et al., 2021^31^	SR-MA	Multi-country	63.1	33.8	8.5	–	Prevalence from 15 study meta-analysis
Choi et al., 2017^33^	CSS	Korea	–	94.1	36.3	–	12-month prevalence
Sun et al., 2017^34^	CSS	China	83.4	76.2	24.1	7.8	12-month prevalence

CSS, cross-sectional; SR-MA, systematic review and meta-analysis; OS, observational study; Op, Opinion; Scop, Scoping review; NR, Narrative review.

Unsurprisingly, non–physical violence was reported at rates two to ten–fold higher than physical violence. However, the prevalence of physical violence is reported as high as 65%^20^ and there is evidence it has increased significantly over the last 30 years relative to the change in non–physical violence.^21^

Labor statistics show workers in the healthcare sector are at far higher risk of experiencing WPV and injury when compared to other risky industries and clinicians perceive violence occurring more commonly.^27^ This reality is becoming the subject of significant public attention which is increasing pressure on healthcare organizations and policy makers to take action (Panel 2).^15^^,^^20^^,^^35^^,^^36^ At the height of the COVID–19 pandemic, tensions between the public and the healthcare system escalated rapidly due to the number of ill patients, the burden of non–pharmaceutical public health interventions and wide dissemination of misinformation.^37^ This likely contributed to a rise in rates and severity of WPV. For example, Brigo et al.^38^ in a study of 235,794 patient encounters, reported that the incidence of attacks in an Italian Emergency Department (ED) increased nearly 86 fold, from 0.05/1000 attacks per month (p = 0.018), to 4.3/1000 attacks per month (p = 0.005) between January 1, 2017 and August 30, 2021.

*Panel 2* **High rates of WPV in the healthcare sector due to unsafe workplaces.**

**The industry policy challenge of WPV in the healthcare sector**

• U.S Healthcare workers experience WPV at a rate almost four times that of other industries (7.8 per 1000 workers).^35^
• Almost 3 in 4 non-fatal workplace injuries involved HCWs or social workers.^36^

• Rates of physical violence have increased^15^^,^^20^ and injuries associated with violent incidence are occurring more regularly.^36^

### Healthcare worker (HCW) underreporting of workplace violence (WPV)

Obtaining accurate and fulsome data on WPV remains challenging. Historically, reporting of WPV has been deficient due to organizational and HCW related barriers with multiple studies identifying reporting rates from 20% to 50%^39^, ^40^, ^41^, ^42^ and one study finding underreporting rates above 89%.^43^ Multiple barriers to WPV reporting have been identified in the literature^44^^,^^45^ and can be categorized as organizational and HCW related (Table 6).

**Table 6:** Barriers to reporting workplace violence.^18^^,^^39^, ^40^, ^41^, ^42^^,^^44^^,^^45^

Organizational	Healthcare workers
Healthcare reporting culture	Expectations of WPV as part of the job
Lack of management accountability	Lack of physical injury
Lack of management support	Lack of time to report
Poor reporting infrastructure and processes	Fear of negative consequences from superiors and perpetrators
Ambiguity in definitions of reportable offences	Unfamiliarity of reporting methods
Complexity of the legal system	Futility due to inaction by organization
Primacy of customer service	

Previous interventions to increase WPV reporting have targeted HCWs, institutions and healthcare systems. The interventions range from providing dynamic risk assessments and enhanced reporting mechanisms to department and jurisdiction–wide voluntary and mandatory guidelines.^29^^,^^46^ It is unclear to what extent regional and institutional initiatives have impacted reported rates, incidence and prevalence of WPV. Arnetz et al.^43^ found that the reported rates of WPV tend to increase when interventions are undertaken due to heightened awareness amongst participants. Further investigation of the efficacy of reporting interventions and the relationships between these interventions and rates of WPV is warranted.

### Impacts of workplace violence (WPV) on healthcare workers (HCWs)

A synopsis of the 39 included articles that report impacts of WPV (Table 7) shows a majority of studies define qualitative impacts with only a small number seeking to quantify and correlate incidents and their impacts. For the purpose of this review, the impacts are categorized as either HCW impacts (those that directly affect the wellbeing of healthcare workers) and patient/healthcare system impacts (those that affect the operation of the healthcare system and patient care). A wide range of impacts are reported spanning multiple domains within each category and with statistically significant associations between domains.^47^ Burnout, stress and mental health impacts are commonly referenced^48^, ^49^, ^50^ with a majority of studies identifying multiple impacts. Suicide, serious injury and even death are reported with grave frequency.

**Table 7:** Synopsis of studies reporting impacts of workplace violence on healthcare workers.

Study, year (reference)	Healthcare Worker Impacts									Patient/healthcare system						No. impacts reported
	Burnout	Stress	Mental health	Self-harm/suicide	Emotional impacts	Family/social	Sleep quality	Physical injury/death	Physical symptoms	Quality of care	Job satisfaction	Job performance	Time away from work	Intent to leave/turnover	Patient-HCW relationship	
Pan et al., 2015^8^								X								1
Kumar et al., 2016^15^	X				X	X				X						4
Duan et al., 2019^16^					X						X			X	X	4
Nowrouzi-Kia et al., 2019^17^			X								X					2
Byon et al., 2021^18^					X	X		X		X	X		X		X	7
Aljohani et al., 2021^19^										X			X			2
Civilotti et al., 2021^20^										X						1
Ramzi et al., 2022^22^	X	X					X				X	X				5
Lei et al., 2022^23^											X	X		X		3
El-Zoghby et al., 2022^24^	X	X	X				X					X				5
Vento et al., 2020^25^	X		X					X		X	X	X	X	X		8
Caruso et al., 2022^26^	X		X					X		X						4
Iacobucci, 2022^27^	X													X		2
Dopelt et al., 2022^28^	X		X		X				X		X			X		6
Viottini et al., 2020^29^			X		X								X	X		4
Liu et al., 2019^30^	X	X					X	X			X	X				6
Tian et al., 2021^31^										X						1
Phillips, 2016^39^	X									X	X	X	X			5
Liu et al., 2020^41^					X	X			X	X				X		5
Nyberg et al., 2020^47^			X						X				X			3
Shaik et al., 2020^48^	X		X								X	X				4
Dye et al., 2020^49^		X	X	X												3
Gimenez et al., 2021^50^	X				X							X				3
Khan et al., 2021^51^			X													1
Hokee et al., 2022^52^	X	X	X	X	X						X					6
Wang et al., 2020^53^		X	X													2
Ghareeb et al., 2021^54^					X					X	X					3
Tiesman et al., 2022^32^	X	X	X		X						X			X		6
Estryn-Behar et al., 2008^55^	X													X		2
Dyrbye et al., 2022^56^	X		X								X			X		4
Sun et al., 2017^34^	X	X					X					X		X		5
Zhang et al., 2018^57^		X					X									2
Havaei et al., 2020^58^	X	X	X	X			X									5
Jakobsson et al., 2020^59^		X	X		X	X	X		X			X		X		8
Dagnaw et al., 2021^60^	X	X							X		X			X		5
Alhamad et al., 2021^61^			X						X			X	X	X		5
Chakraborty et al., 2022^62^		X	X		X		X				X				X	6
Farrell et al., 2014^63^					X								X			2
Bryant-Genevier et al., 2021^64^			X							X		X	X	X		5

The reporting methods and outcomes vary widely across studies with the majority utilizing retrospective self–reporting techniques with significant definitional variation. Although this makes reliable comparison and synthesis difficult, consistent trends have emerged, showing WPV likely contributes significantly to poor mental and physical health in victims. Fang et al.^65^ found that 71% of HCWs surveyed in Northern China who experienced physical violence reported depressive symptoms, while 51% of 477 doctors in India reported depressive symptoms, anxiety and stress, and 52% reported a loss of self–esteem and shame associated with WPV.^66^ Kumari et al.^40^ report that two thirds of HCWs experienced violence resulting in physical injury, often requiring temporary or even permanent leave from work. Khan et al.^51^ found around two thirds of HCW exposed to violence suffered mental health consequences and Hokee et al.^52^ found that paramedics who experienced WPV had higher levels of stress and anxiety. Similarly, Wang et al.^53^ found that 38% of Chinese doctors reported mental health issues associated with WPV and Ghareeb et al.^54^ found 84% of Jordanian HCWs reported negative psychological impacts due to violence experienced over the COVID–19 pandemic. A large study of more than 26,000 public health workers (non–clinical and clinical) in the U.S.^32^ found WPV prevalence ratios (adjusted for confounders) for depression symptoms (1.21, 95% CI = 1.15, 1.27), anxiety (1.21, 95% CI = 1.15, 1.27), PTSD (1.31, 95% CI = 1.25, 1.37), and suicidal ideation (1.26, 95% CI = 1.14, 1.38), suggestive of causal relationships between WPV and long term mental health impacts.

The relationships between WPV and HCW burnout has been assessed in a number of large cross–sectional studies. An extensive 2008 study^55^ of more than 39,000 nurses from ten European countries found that HCWs who experienced WPV on a monthly (OR; 1.38, 95% CI = 1.26, 1.52 p < 0.001) or weekly (OR; 1.90, 95% CI = 1.72, 2.11 p < 0.001) basis had higher odds of experiencing burnout. A 2022 study^56^ of 2450 physicians in the U.S. found those who experienced mistreatment or discrimination which occurred weekly or several times per year, had increased odds ratios for burnout of 1.70 (95% CI = 1.38, 2.08) and 2.20 (95% CI = 1.89, 2.57), respectively.

Multiple studies have quantified the correlations between WPV and various impacts. In a large cross–sectional series (n = 2617) Sun et al.^34^ found that WPV was positively correlated with psychological stress (r = 0.382, p < 0.001) and negatively correlated with sleep quality (r = −0.281, p < 0.001) and subjective health (r = −0.471, p < 0.001). A similar study^57^ of a nursing population in China (n = 1024) showed psychological stress was positively correlated (β = 0.295, p < 0.01), and sleep quality (β = −0.198, p < 0.01) and subjective health (β = −0.252, p < 0.01) negatively correlated with WPV. Havaei et al.^58^ found that in a population of surgical nurses in Canada (n = 537), WPV was positively correlated with musculoskeletal injuries (r = 0.33, p < 0.01) and anxiety (r = 0.44, p < 0.01).

### Impacts of workplace violence (WPV) on patient care and costs to the healthcare system

The impacts of WPV on HCWs and the associated direct and indirect costs to the healthcare system have the potential to put patients at risk. A number of studies report a reduction in quality of care and an increased risk of medical errors resulting from WPV.^17^^,^^30^^,^^57^^,^^59^^,^^60^^,^^65^ Alhamad et al.^61^ in a survey of physicians in Jordan, reported 72% of respondents felt their job performance was affected by WPV. While in a systematic review of 36 studies, Guo et al.^67^ identified self–reported delays or omissions in testing, increases in post–operative complications, higher perceived adverse events and decreased patient safety. Dyrbye et al.^56^ found WPV was positively correlated with burnout and the burnout was associated with higher odds of perceived major medical errors.

Loss of staff has been linked to WPV in multiple studies.^16^^,^^17^^,^^24^^,^^30^^,^^34^^,^^58^^,^^62^ Burnout associated with WPV has been implicated in high rates of attrition and intention of HCWs to leave their current employers.^56^ In China, Liu et al.^41^ reported 50% of operating room nurses intended to quit or change their career due to WPV and 60% of new nurses quit within the first six months of employment. Additionally, the authors estimate the cost of replacing a nurse to be $88,000 USD and the total cost of replacing nurses up to $4 billion USD per year.^41^ This is particularly concerning in the context of a global shortage of HCWs, with the WHO estimating a ten million health worker gap by 2030.^68^

Aljohani et al.^19^ report WPV resulted in 14.7 days away from work per 10,000 workers for hospital employees as compared to 2.8 days for non–governmental, non–HCWs. The direct costs associated with prevention of violence are also staggering. Grossman and Choucair^35^ report that $4.7 billion USD was spent on hospital security in the U.S., with an estimated 18% ($847 million USD) directly attributed to workplace violence prevention efforts.

Work absence was another common impact reported by multiple authors.^29^^,^^39^^,^^47^^,^^56^^,^^59^^,^^63^ Viottini et al.^29^ found in one series from a large Italian teaching hospital, that 53% of workers who experienced WPV required time–off work. Similarly, Dyrbye et al.^56^ reported that in 20% of 13,000 cases of WPV resulting in injury, the victim required three to five days away from work. Nyberg et al.^47^ in a systematic review, found statistically significant associations between physical WPV and work absence in three of four included studies.

In addition to direct costs, multiple authors identify productivity losses as a consequence of WPV.^15^^,^^39^^,^^64^^,^^67^ Guo et al.^67^ identified losses ranging from 9.5% to 22.1% with an estimated value of $1484–$11,581 per nurse, per year.

## Prevention and mitigation interventions

### Historical development and assessment of interventions

Interventions to prevent, mitigate and manage WPV have been the focus of much discussion and research for a number of decades. In 2004, the U.S. National Institute for Occupational Safety and Health (NIOSH) identified major barriers to successful prevention and mitigation of WPV.^69^ Many of the barriers remain stubbornly unchanged, or have worsened, including a hostile or toxic organizational culture, lack of awareness of the extent of WPV, culture of violence in the community, lack of worker empowerment, lack of training, lack of information and lack of WPV reporting.

Developing and implementing effective interventions, given the array of causative factors and varied impacts of WPV, has proven difficult. A wide range of initiatives addressing many aspects of WPV have been studied, however training interventions focused on nursing staff appear most frequently in the literature. Historically, WPV intervention studies suffer from methodological problems including lack of statistical power, control groups and self–reporting.^70^ A 2022 systematic review of 17 studies by Kumari et al.^71^ including randomized control, pre–post and longitudinal methods, found evidence that some interventions improved HCWs perceptions of dealing with factors that lead to WPV, however, most of the included studies were small with significant methodological challenges.

There is little evidence supporting the long–term efficacy of interventions. However, some demonstrate positive outcomes in reducing risks and giving HCWs greater confidence in managing WPV. Arnetz et al.^43^ in the largest randomized control study, enrolled 41 nursing units (totaling 2863 staff) and assessed the impact of a range of violence reduction strategies. The authors found significant variation in the design of these strategies at a unit level, ranging from better coordination between disciplines to customer service training to panic alarms and lighting upgrades. The study demonstrated short–term reductions in violent incident rates and longer–term reductions in violence–related injuries and risks. Baby et al.^72^ concluded that communication skills and mindfulness interventions showed a decrease in perceived aggression and distress at six months post intervention, and a patient handover tool that focused specifically on identifying and discussing how to manage aggressive patients^73^ improved HCW's reported feelings of safety.

A range of other novel interventions appear in the literature with, at best, variable efficacy. A single hospital trial of body worn cameras in a psychiatric unit^74^ demonstrated that the use of restraints halved and assaults reduced in three of five units. However, verbal violence during the trial increased. In another trial in which nurses flagged individuals for video surveillance if they were deemed at risk of violent behavior, showed that less than 1% displayed such behavior.^75^

### Organizational, legal and regulatory interventions to stem workplace violence (WPV)

Organizational level management of WPV is investigated primarily through cross–sectional survey studies providing HCW perceptions about the efficacy of counter interventions. The results suggest that HCWs believe healthcare organizations could do more to prevent WPV. Kumar et al.^15^ found 78.9% of respondents believe incidents against HCW are preventable. While Dopelt et al.^28^ found 31% of HCWs felt management only dealt with violence to a limited extent.

Regulatory and legislative interventions are becoming more common, but often they receive only cursory attention in the literature with few studies assessing their full impact. Numerous policies and legislative measures have been implemented in the past two decades to address WPV across Europe, the U.K., U.S., Australia and a number of Asian countries. Recently (September 2023) a bipartisan bill was introduced in the U.S. Senate to create federal criminal offences relating to WPV against HCW, which follows laws enacted in nearly 40 U.S. states over the preceding years.^3^ EU members introduced country–specific legislation derived from the common workplace safety guidelines and in 2007 an EU framework agreement confirming the duty of employers to protect healthcare workers was signed.^29^ In India,^25^ the Philippines^11^ and the U.S.^76^ recent legislative actions have introduced new reporting obligations for organizations to protect their healthcare workers or to create specific criminal offence categories and/or increase penalties for offenders. There is some evidence that increased workplace safety obligations may reduce violent incidents.^77^ However, even where standards are mandated, there is a wide interpretation and varied implementation which creates inconsistency in application and challenges in the external generalizability of the interventions.^78^

### What healthcare workers (HCWs) say: recommendations from the victims’ perspective

HCWs have recommended a range of actions to curb WPV. The most common suggestions include increased security, staffing levels, training and worker support, and improved infrastructure.^15^^,^^23^^,^^25^^,^^26^^,^^32^^,^^41^ Patient education to manage expectations of their care^26^ and engagement in public messaging campaigns have also been proposed.^25^ Other, more punitive measures, have also been suggested including refusal of care to violent offenders and removing patients from primary care patient registers.^21^^,^^27^

### Potential tools to understand the contextual gaps and impacts on healthcare workers (HCWs)

It is clear from the literature that the local workplace context and culture have significant impacts on the factors that contribute to WPV. Context is comprised of a range of elements including patient/visitor characteristics, HCW characteristics, physical environment, organizational, economic and socio–cultural factors. No single intervention has demonstrated compelling evidence for efficacy across different contexts, and there appears to be a lack of robust assessment of the specific internal and external drivers for WPV at individual healthcare facility levels.

Our literature search failed to identify a comprehensive framework for assessing an organization's dynamic WPV risks across multiple contextual elements. Such a framework could aid organizations in identifying and prioritizing areas where further investigation is required, and where interventions are more likely to provide the greatest impact on WPV reduction.

## Discussion

Our review emphasizes the general consensus that violence against HCWs is a critical and growing concern due to its widespread detrimental impacts on HCWs, healthcare systems and society as a whole. The persistent nature of the problem, despite increasing awareness and ongoing interventions, highlights the complexity of effectively addressing and mitigating WPV. The interplay between workplace, patient and socio–cultural factors creates a wicked set of challenges for individual healthcare units and hospitals.

Our review found sufficient evidence to confirm WPV contributes to poor mental and physical health of HCWs. There is also evidence of a causal relationship between these WPV impacts and poorer quality patient care, and increased direct and indirect costs to healthcare systems. This highlights the importance of addressing WPV, particularly in the context of a growing shortage in healthcare workers and resources. The consequences HCWs bear as a result of WPV contribution significantly to staff turnover which reduces access to care, particularly for vulnerable populations, and can further exacerbate patient aggression.

The authors believe there is an important role for legislative action against WPV despite finding little formal evidence for its impact. Legislative action should be targeted at disincentivizing violence and aggression toward HCWs, appropriately allocating responsibility for safety to organizational stakeholders and raising public awareness through advocacy and educational campaigns. Any legislative action that creates additional criminal penalties for violence against HCWs should aim to prevent, rather than mitigate the impacts violence and the rights of perpetrators should be considered to maintain the principle of health equity. However, it should be acknowledged that healthcare environments are unique in that violent behavior can have a direct and immediate impact to the safety and care of multiple vulnerable stakeholders simultaneously.

The evidence points to a knowledge–action gap in the way healthcare organizations assess the risks of WPV. We propose an actionable framework which can aid administrators and managers in assessing the unique characteristics of their WPV risks. This framework is designed to incorporate multiple stakeholders and contextual elements in an objective manner while maintaining the human element of medical care that is so critical to promoting safe work environments.

**Fig. 2:**
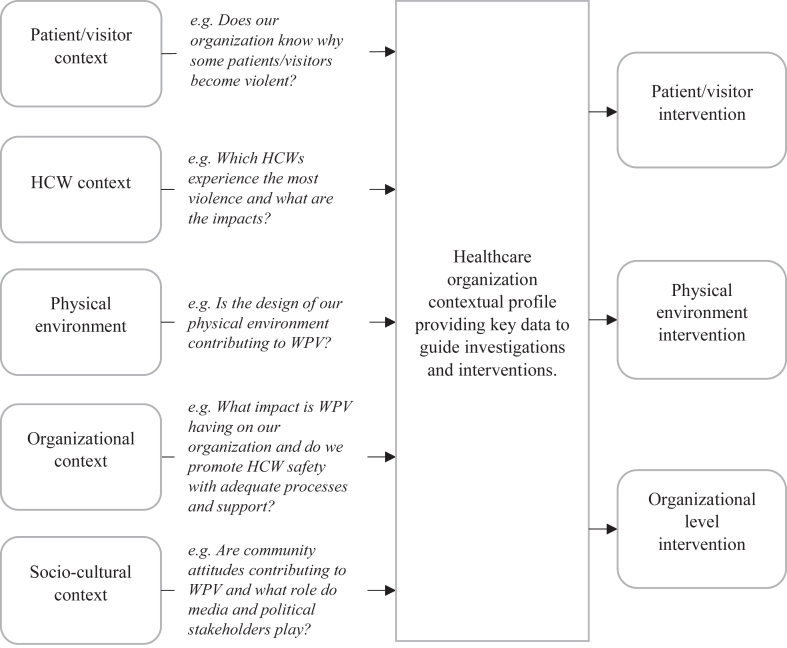
**Conceptual framework for healthcare workplace contextual profile**.

The framework calls for an assessment of an organization's WPV risks across multiple contextual elements including the patient/visitor characteristics, HCW characteristics, built environment, organizational and socio–cultural characteristics. Developing this contextual profile for an individual organization should involve a range of stakeholders including HCWs, security, administration, management, regulators, policy makers and patients/visitors. This contextual profile compliments existing standards and guidelines, such as the U.S. Occupational Safety and Health Administration (OSHA) guidelines for preventing workplace violence,^79^ by integrating both internal and external factors that contribute to WPV at a specific healthcare facility or unit. A conceptual framework for developing an organizational risk profile, including examples of priming questions, is provided in Fig. 2.

The success of any intervention in combating WPV is dependent on accurate and truthful data. The need for reliable data related to the incidence, prevalence, causation and impacts of WPV cannot be overstated. Effective interventions can only be developed if the scope and extent of the problem is better defined and regularly measured. Hospital administrators and policy makers should address this gap more fulsomely and accurately by capturing WPV data at a system–wide level. The authors suggest jurisdictional or nation–wide reporting obligations be considered at all healthcare facilities with incentives built into management and accreditation processes. An example of progress in this area is the implementation of WPV prevention standards for all hospitals seeking Joint Commission accreditation.^80^

A core element in robust WPV reporting is active participation of HCWs. Common definitions of reportable incidents are needed, reporting processes should not be burdensome and HCWs must feel empowered and safe through management actions. Barach and Small^81^ describe a number of disincentives to adverse event reporting across a range of industries which bear relevance to WPV. Organizational culture, time and effort, skepticism, lack of trust, fear of reprisal, and ineffectiveness were identified as common disincentives to reporting across a range of industries. Addressing these factors will be critical in implementing WPV reporting obligations at an organizational level.

Another important area of intervention is advocacy by healthcare stakeholders, policy makers and the media. It is clear that negative attitudes towards the health system, and those who work in it, play a role in enabling WPV. The public's attitudes are impacted by the actions of both policy makers and the media and ensuring objective input from healthcare stakeholders in public discourse is vital.

Our review has several limitations. Firstly, narrative reviews are not intended to assess the quality of the literature analyzed. A majority of the studies are retrospective and rely on self–reporting of incidents and harm. This makes an accurate risk assessment across populations and timeframes difficult. Second, the literature is skewed toward understanding causation from the victim's perspective with an absence of data from the aggressor's perspective. This may distort the data which could undermine the efficacy of interventions. Third, given the limitations of research into WPV, the wide variety of contributory factors and healthcare contexts, there is no consensus or evidence base to prioritize interventions. Finally, the contextual profile presented in this paper has yet to be formally tested and will require application in real–world settings to understand its utility and sustainability. Despite these limitations this review provides a comprehensive overview of the existing research and clearly identifies key themes and challenges to help guide future research and interventions.

Workplace violence against healthcare workers is never acceptable, moral or legal and should not be tolerated. Current mitigation and prevention efforts through training, behavioral cues, facility design and operational policies have mostly fallen short. Organizations could benefit from sustained professional pressure to better define the factors that contribute to WPV and the risk mechanisms by which to assess the unique characteristics and impacts of WPV on their organizations.

WPV is driven by a complex interaction of internal and external factors. Defining how these factors interact and contribute to WPV is important in maximizing the impact of organizational and jurisdictional level investigations and interventions. Integrating the profile of an organization's risks into management decision making, reporting and accreditation will assist in prioritizing the activities with the greatest impact on reducing and mitigating WPV. Our review highlights the growing problem of WPV and the urgent need for action by multiple stakeholders, improved and more transparent reporting and funding of further research into suitable outcome metrics and effective mitigation strategies.

## Contributors

CO: methodology, literature review and original draft author. AvZ and PB: conceptualization, oversight, review and editing. All authors confirm they had full access to all data in the study and accept responsibility to submit for publication. All authors contributed to the article and have approved the submitted version.

## Declaration of interests

The authors declare the research was conducted in the absence of any commercial or financial relationships that could be construed as a potential conflict of interest. No funding was received to undertake this review.

Conor O'Brien is an unpaid board member of the not–for–profit support service organization, Queensland Homicide Victims Support Group (QHVSG). QHVSG has no direct role in activities related to preventing or managing violence against healthcare workers however may provide support services to loved ones of healthcare workers who have been victims of homicide.
