# Supplementary material for: The growing burden of workplace violence against healthcare workers: trends in prevalence, risk factors, consequences, and prevention – a narrative review
Source: eClinicalMedicine. 2024 May 27;72:102641. doi: 10.1016/j.eclinm.2024.102641 (PMC11152903; doi:10.1016/j.eclinm.2024.102641)
Supplement: Supplementary Table S1 [file mmc1.docx]

**Supplementary Table 1**

Table 1 - Included study characteristics

| **Ref.** | **Type** | **Primary outcome(s) / Purpose** | **Incidence / Prevalence / Outcome** | **Causes and risk factors for violence** | **Setting** | **Perpetrator characteristics** | **Impact on staff** | **Impact on healthcare system** | **Prevention and remediation strategies and efficacy** |
| --- | --- | --- | --- | --- | --- | --- | --- | --- | --- |
| American College of Emergency Physicians, 2022^1^ | News report | Violence in American EDs for past five years. | 85% of emergency physicians believe the rate of violence experienced in emergency departments has increased over the past five years, with 45% indicating it has greatly increased. | Mental health, drug seeking, intoxication. Lack of punitive consequences. | American EDs | N/A | Reduced patient care, HCW burnout, mental health impacts. | Loss of productivity, poor patient care. | Awareness campaign. |
| Massachusetts Health and Hospital Association, 2023^2^ | Report | Quantify the level of WPV against HCW and consequences. | Every 38 minutes someone in Massachusetts healthcare facility experiences violences. A significant increase in WPV on previous years. | N/A | Massachusetts Healthcare system | 91% patients. | Trauma, attrition, patient care. | Reduced resources and poor patient care. | Comprehensive approach including training, awareness, reporting, patient/visitor engagement, legislative changes. |
| Gamble, 2023^3^ | News report | Introduction of Bill into U.S. senate. | N/A | N/A | U.S. hospital system | N/A | N/A | N/A | Legislative action. |
| U.S. Bureau of Labor Statistics, 2020^4^ | Factsheet | Incidence of WPV. | Non-fatal WPV incidence 10.4 per 10,000 HCWs. | N/A | U.S. healthcare system. | N/A | N/A | N/A | Not reported. |
| Eala et al, 2022^5^ | Correspondence | Since 2017, at least ten doctors have been violently killed in various regions of the Philippines | Not reported. | Not reported. | Philippines healthcare system. | Not reported. | Feeling less secure resulting in loss of resources within the workforce. | Loss of HCWs, particularly from vulnerable, underserved areas. | Legislative action to increase penalties for perpetrators. |
| Lorettu et al, 2021^6^ | Retrospective data analysis | Characteristics of homicides against HCWs. | 21 doctors killed in 30 year period. | Primary reason for homicide was revenge, often preceded by stalking. Medical malpractice allegations involved in a number of cases. | Italian healthcare system. | Most often regular patients, however can also be occasional patients or loved ones. Psychiatric disorders, effects of substances are mentioned. | Not reported. | Not reported. | Establish conflict mediation system for medical malpractice allegations. Specific violence risk assessment for psychiatric patients. Training in communication skills for physicians. |
| Kelly, 2022^7^ | News report | Report on violence against HCWs in conflict zones. | 161 medics killed, 320 wounded and 170 kidnapped. | Not reported. | Conflict zones in Europe, Middle East and Africa. | Not reported. | Deaths and injuries. | Loss of health infrastructure and personal. Impact on public health programs. | Government intervention for stronger deterrence and prosecution. Military leaders to review and reform practices. |
| Pan et al, 2015^8^ | Review article | Collection of evidence of serious violence against medical practitioners in China. | 101 serious incidents of medical violence with 24 doctors or nurses being killed. | Dissatisfaction with treatment and services and fees. Distrust of medical profession, media reporting. | Tertiary hospitals experienced the highest proportion of serious incidents. | Not reported. | 101 incidents of serious medical violence occurred in the past decade, in which 24 doctors or nurses died. | Not reported. | Zero-profit policy for medications, increased incomes for medical staff, judiciary for medical disputes, increasing punishment for WPV, improvement of communication skills. |
| Kumar et al, 2016^15^ | Cross-sectional survey | Prevalence of WPV among doctors and associated risk factors. | 47.02% of the doctors reported having an experience of violence. Females more than males. verbal violence most common followed by emotional and physical violence. | Younger doctors with less work experience. Long waiting times and delays in care. Commercialization and increasing out of pocket expenditure. Increasing access of information by patients and the ability to pursue legal avenues against doctors. | Multiple medical and surgical departments in a tertiary care hospital in Delhi, India. | Patients and family members. | Psychological and physical well-being, quality of health care and health organization as a whole. Burnout, low self-esteem and self-destructive aggression. | Reduced resources due to impact on HCWs. | 78.9% participants believed that these incidents could have been prevented. Lack of training cited by a number of respondents. Suggested measures included; increasing clinical and security staff, restricting visiting hours, improving communication, proper compliance with the standard operating procedures, vigilant management and proper infrastructure. |
| Duan et al, 2019^16^ | Cross-sectional survey | Prevalence of workplace violence against physicians. Examine the association between WPV and job satisfaction, job burnout and turnover intention. | 66.19% (832/1257) of participants reported having experienced WPV within the past 12months. Verbal violence 65.3%, physical violence and sexual harassment toward physicians was 12.57% (158/1257) and 0.88%. Prevalence among medical staff ranges from 50% to 88%. | Not reported. | Hospitals across multiple jurisdictions in China. | Patients’ relatives (54.2%), patients (26.4%). | Reduced job satisfaction, emotional exhaustion turnover intentions and lower job satisfaction. Breakdown of trust between doctor and patient. | Not reported. | Focus on increasing social supports, establishment of "code green" response for violence, and policy and legislative support. |
| Nowrouzi-Kia et al, 2019^17^ | Systematic review and meta-analysis. | Types and prevalence of WPV among doctors and factors that cause WPV. | 69% for all types of violence. | Patient factors; Intoxication and behavioral / mental health issues, expectations of care not being met. Environmental factors; Working in remote care areas.  Organizational factors; increasing demands on healthcare systems, insufficient security, lacking preventative measures, lack of management support, absence of organizational policies, inadequate staffing, long wait times. Clinician factors; lack of communication skills, lack of training, lack of experience and higher workloads. | Range of settings including hospital and outpatient. | Not reported. | Poor mental health, unhealthy coping mechanisms, reduced job satisfaction. | Higher rates of error and poorer patient outcomes and physical violence resulting in reduced doctor retention. | Interventions focused on dealing with mental health impacts of WPV. |
| Byon et al, 2021^18^ | Cross-sectional survey | WPV during the COVID-19 pandemic and attitudes to reporting. | 44.4% of respondents reported experiencing physical violence and 67.8% verbal abuse. | Unmet patient needs, staff shortages, insufficient time and patient support, and decreased capacity. Nurses providing care to COVID-19 patient's were more likely to experience violence and verbal abuse. COVID-19 specific factors include patient fear of COVID-19 prognosis, stress from quarantine, emotional/behavioral changes, panic, misplaced anger, misinformation. Nurses suffered from anxiety, work pressures, staff shortages, lack of peer and organizational support. Underreporting caused by belief that violence is part of the job, unstandardized reporting process, ambiguity on the reportability of violence, including an unclear definition of violence, and lack of management support. | Hospital environment; majority emergency department, intensive care and medical-surgical units. | Not reported. | Physical injury, psychological and emotional impacts, work functioning (e.g., sick leave, job satisfaction), relationship with patients, quality of care, social/ general (e.g., disturbed social and family life). | Financial (e.g., treatment cost, loss of work time) aspects of health and well-being. | Identification of risk factors, increase reporting, more stringent legislative requirements to protect healthcare workers. |
| Aljohani et al, 2021^19^ | Systematic review and meta-analysis | Incidence and causes of WPV. | 9072 cases of WPV, 72% verbal abuse, 18% physical abuse, 9.5% other types eg sexual harassment and stalking. | Based on Occupational Safety and Health Administration (OSHA) identified risk factors; drug and alcohol abuse, history of violence and psychiatric illnesses, long wait times, understaffed EDs and unrestricted movement of the public. | Emergency Department. | Not reported. | Increased days away from work. | U.S BLS; 14.7 days away from work per 10,000 workers for hospital employees and 35.3 days away from work per 10,000 workers for nursing and residential care facilities. Decreased productivity and ultimately a decrease in the quality of patient care. | 24 hour security, violence prevention workshops for staff, improved communication and increasing staffing, supportive policy and legislation. |
| Civilotti et al, 2021^20^ | Scoping review and narrative synthesis | Incidence and prevalence of WPV. | verbal 11.9% to 93.3%, physical 0% to 53%. Career prevalence ranges from Verbal 48.8% to 90.9% and Physical 25.7% to 64.65. | Misunderstandings or disputes regarding medical issues, dissatisfaction with treatment or physicians, physical contact during the provision of care, enforced personal care and enforced medical treatment, long waits, perception of quality of care. | Most commonly emergency department and psychiatric wards. | Patients primary aggressors. Aged 30-50. Abnormal mental states and cognitive impairment. | Impact workers and families leading to poorer patient care. | Poor patient care. | Effective strategies must involve monitoring tools and diverse and multidisciplinary working groups. Situational factors appear to be those which best predict violence however they cannot be addressed in isolation to mitigate violence. |
| Spelten et al, 2022^21^ | Focus groups - descriptive qualitative design | Identify and discuss strategies used by prehospital emergency health care workers, in response to violence and aggression from patients and bystanders. | Not reported. | Not reported. | Pre-hospital environment attended by paramedics in Canada. | Not reported. | Not reported. | Not reported. | Training, refusal of care, public campaigns for prevention, flagging violent offenders. |
| Ramzi et al, 2022^22^ | Systematic review and meta-analysis | Prevalence during the COVID-19 pandemic. | Total prevalence 47%. Physical violence 17%. Psychological violence 44%. | Insufficient number of medical staff and unmet patient demands, acute illness, fear of unpredictability, and severe stress. | Multiple hospital settings. | Not reported. | Decreased job satisfaction and productivity, low quality of life, increased stress, burnout, and sleep disorders. | Productivity and turnover. | Not reported. |
| Lei et al, 2022^23^ | Cross-sectional survey | Prevalence, characteristics, and predictors of workplace violence (WPV). | 79.39% of nurses exposed to violence in previous 12 months. 78.38% non-physical, 39.65% physical.  Verbal abuse (75.22%), threat (51.51%), physical assault (37.40%), verbal sexual harassment (24.81%), physical sexual assault (12.19%). | Male, bachelor's degree, working in central China. Unmet patient needs, long waiting times, drug and alcohol and self-perceived high medical costs. | Emergency Department. | Patient's relatives main perpetrators, less violence occurs with better health insurance reimbursement, higher patients’ medical literacy and education level. | Reduced job satisfaction, poor work performance, high nurse turnover and poor quality of life. | Not reported. | Training, hardening infrastructure in the ED, effective triage to reduce wait times and ensuring appropriately experienced staffing for high risk times e.g. night shifts. |
| El-Zoghby et al, 2022^24^ | Cross-sectional survey | Prevalence of WPV and negative health impacts. | 26% experiencing shouting/yelling every day. 15.8% reported having objects thrown at them once a month. 84% of medical trainees reported being subjected to mistreatment in clinical settings. | Working night shifts, longer duration of shifts, type of physician specialty (ED, ICU, Anaesthetics). Heavy workloads, low physician–patient ratios, unmet expectations of patients and stressful work climate, negative attitudes of institutional managers, the COVID-19 pandemic, inadequate security, dissatisfaction with service, shortage of staff and violence portrayed in the media. | Various hospital settings. | Staff members (59.4%), with Patient's relatives second (57.4%) and patients fourth (24.8%). | Anxiety and depression in 59.4% of respondents, poor sleeping amongst 86.1%. Lower levels of confidence, attrition, impaired performance, stress, psychosomatic symptoms, depression, burnout, and drug abuse. | Violence generates stress and stressed physicians are more susceptible to violence. | Not reported. |
| Vento et al, 2020^25^ | Opinion | 181 NHS Trusts in England reported 56,435 physical assaults on staff in 2016–2017. | N/A | Working in remote health care areas, understaffing, emotional or mental stress of patients or visitors, insufficient security, and lack of preventative measures. Insufficient time devoted to patients, poor communication, long waiting times and overcrowding in waiting areas Lack of trust in HCWs or in the healthcare system, dissatisfaction with treatment or care provided and unrealistic expectations of patients and families over treatment success. Negative media reporting also mentioned. | Emergency Departments, Mental Health Units, Drug and Alcohol Clinics, Ambulance services and remote Health posts. | N/A | Deaths or life-threatening injuries, reduced work interest, job dissatisfaction, decreased retention, depression, post-traumatic stress disorder, decline of ethical values, increased practice of defensive medicine. | Burnout, more leave days, impaired work functioning, lower patient safety, and more adverse events. | Harsher penalties for perpetrators however unlikely to solve the problem alone. Noting lack of evidence of efficacy of measures. |
| Caruso et al, 2022^26^ | Narrative review | Incidence across various global regions, impacts, mitigation strategies. | Healthcare professionals (mainly nurses) have a 16 times increased risk of violence as compared to workers in other sectors and are four times more likely to require time away from work as a result of violence. Germany, data show that about 90% of 831 doctors had faced some form of aggression in their career and about 70% in the previous year. | Cultural factors, prevention and control measures associated with COVID-19, lack of communication skills and empathy. Younger age, inexperience, female gender, lack of training to de-escalate, poor communication, history of violence, drugs and alcohol, confusion states, lack of resources and of staffing, poor workplace design, lack of support for staff, burnout, lack of violence-prevention programs. | Multiple in-patient settings. | Not reported. | Somatic injuries, but also with psychological consequences, such as burnout, post-traumatic stress disorder, depression and anxiety. | Higher rates of medical errors and poorer quality of care. | Education, development of safety standards, timely response after violence occurs. Better infrastructure design, better reporting and HCW support have less violence. Promoting behavioral changes in HCW, debriefing, regular follow-up sessions, and psychological support. |
| Iacobucci, 2022^27^ | News report | N/A | Near doubling of violent incidents over the past five years. 17 043 prosecutions on the charge of assault of an emergency worker, compared with 13 392 prosecutions in 2020, and 11 257 prosecutions in 2019. | Lack of timely access to healthcare, fewer GPs, false media reports of GPs being closed during the COVID pandemic. | GP and primary health clinics. | Not reported. | Anecdotal reports this is causing some GPs to leave their jobs as well as burnout, demoralization, mental fatigue. | Reduced number of primary care resources including reduced operating times. Cost of damage to premises. | Updating zero tolerance signage in clinics, practices be given greater powers to remove patients immediately from their list of registered patients. Public education campaigns, training to improve safety and wellbeing of staff, legislation increasing penalties for violence, working with prosecutors for more effective prosecution, national data collection, improving training for staff, ensuring mental health support for staff. |
| Dopelt et al, 2022^28^ | Cross-sectional | Incidence during COVID-19 pandemic. | 71% exposed to any kind of violence, 69% to non-physical and 11% to physical. 64% of respondents perceived violence increasing over the pandemic. | Long wait times. anxiety due to COVID-19, lack of hospital resources and the inability to visit critically ill relatives, dissatisfaction with the attitude of the treating staff, bureaucracy, dissatisfaction with the treatment, uncomfortable physical conditions, the effects of alcohol/medications/drugs, communication problems. Lower seniority, working in emergency or internal departments, and being a nurse or a doctor. | Inpatient hospital setting. | Not reported. | Physical and psychological harm, job dissatisfaction, anger, shame, anxiety, sadness, depression, insomnia, burnout, and increased turnover, abandoning the profession. 5% were absent from work following a violent incident, 5% sought emotional support. | Reduction in quality of the healthcare services provided and increased healthcare costs. | More support from management. Additional security, better communication, more staff to reduce wait times. |
| Viottini et al, 2020^29^ | Retrospective observational study | 3-year incidence (2015/17) of WPV through a hospital reporting system and identification of associated risk factors. | 3-year incidence of 363 (3.3%) HCW reporting at least one incident of aggression through a hospital reporting system. Females majority (77.5%) and nurses (64.3%). Verbal 76.9%. | Female, <50 years of age, having worked for 6–15 years. Patients with cognitive impairment, psychiatric disorders and substance abuse. Communication failures and language barriers. Patient expectations, public distrust. | Large tertiary hospital in Italy. | Patient most common aggressor (46.7%), parents (42.3%), colleagues (3%). | Anger, anxiety and anguish, guilt, shame and post-traumatic stress disorder. | Intentions to leave and absenteeism.  53% required work interruption, 11.3% required medical and psychological treatment. | Duty of care by employers, increasing reporting and investigation at a system wide level. |
| Liu et al, 2019^30^ | Cross-sectional survey | Relationship between WPV, nurse outcomes and patient safety. | 28% reported frequent verbal abuse and 96% reported infrequent experience of any type of WPV in past 12 months | Not reported. | 23 hospitals in Southern China | Not reported. | Higher incidences of burnout, less job satisfaction, lower patient safety and more adverse events. | Lower patient safety and more adverse events. | Zero-tolerance policies. |
| Tian et al, 2021^31^ | Systematic review and meta-analysis. | Summarize the evidence on the prevalence of WPV against GPs globally. | 63.1% experienced any form of WPV, 33.8% encountered non-physical violence, and 8.5% reported experiencing physical violence. Physical violence increased to 13% from 7% in the period after 1999.  Prevalence of physical violence has significantly increased over past 30 years while non-physical violence has decreased. | Rates differed based on location, sex and practice setting. Physical violence prevalence increased with study period. | Physical violence was the most prevalent in general hospital settings. | Not reported. | Reduced job commitment, sleep disruption, mental disorder, turnover, and even death. | Care being compromised due to resource availability and quality. | Not reported. |
| Choi et al, 2017^32^ | Cross-sectional survey | Incidence of WPV, staff turnover intention and quality of life. | 95.5% reported that they had experienced WPV in previous 1 year. Verbal violence; 94.1%, Physical threats; 89.9%, Physical violence; 36.3%. | Night shifts, psychiatric units, less experience. | Three hospitals in metropolitan Korea. | Patient's relatives main perpetrators. | Correlation of physical violence on intention to leave. | Loss of resources. | Pre-vocational education particularly focusing on at risk groups. |
| Grossman et al, 2019^33^ | Review article | Review of WPV in the healthcare sector and it's impacts. | Healthcare has highest rates of WPV of all workplaces. 27% of workplace fatalities occurred in healthcare settings. Costs of violence in 2016 estimated at $428.5m USD. 60% of Emergency physicians have been physically assaulted at work. | Drug seeking, intoxication. | U.S. healthcare system. | Most physical assaults committed by patients. | Nurses and physicians have higher rates of suicide than general population. | Lower standards of care, costs from injury and absenteeism, staff turnover, loss of productivity. | Administrators role in identifying patients at high risk of violence, support local violence prevention programs, addressing risk factors, such as mental health and substance abuse, at a primary care level, encourage restriction of access to firearms, better design of facilities and security infrastructure, staff training. |
| Reed et al, 2023^34^ | News report | Assess state of play and impact of WPV on U.S. healthcare system. | 3 in 4 nonfatal workplace injuries involved healthcare and social work. Injuries from WPV in healthcare were up by 62% in 2018 compared to 2011. Trend likely continued upward during COVID-19. 40% of HCW experienced WPV in past 2 years. | Not reported | U.S. healthcare system. | Not reported | Injury and deaths. | Not reported | Identification of high risk individuals, increased training, AI technology to detect firearms, legislative activity to require healthcare providers to implement plans to protect employees, new standards from accreditation authorities. |
| Massaro et al, 2021^35^ | Retrospective study | Impact of COVID-19 on social media discourse. | N/A | N/A | Italian social media landscape | N/A | N/A | N/A | N/A |
| Brigo et al, 2022^36^ | Quasi-experimental interrupted time-series analysis | Effect of COVID-19 on ED attacks on HCWs. | Increase in the rate of monthly attacks on HCWs during COVID-19. | Burden on health resources from pandemic resulting in reduced quality of care, long working hours, increased stress on HCWs. | Emergency department in a general hospital in Southern Italy. | Not reported. | Not reported. | Not reported. | Raising awareness. |
| Phillips et al, 2016^37^ | Review article | Prevalence of WPV, risk factors, and the use of metal detectors in preventing violence. | 78% of ED physicians experienced WPV in past 12 months. | Altered mental status, intoxication, long wait times, poor/uncomfortable environment. | Multiple with ED being a leading area for WPV. | Patients with decompensated mental illness, altered mental status. | Increased rates of missed workdays, burnout, and job dissatisfaction along with decreased productivity and overall feelings of safety among staff members. | Decreased productivity and loss of resources. | No evidence metal detectors reduce rates of violence. Suggested involvement of law enforcement in developing violence prevention as well as focus on staff training. |
| Kumari et al, 2020^38^ | Narrative review | Characteristics, prevalence, risk factors and mitigation strategies for WPV | Wide range quoted 54% (Thailand) to 70% (Morocco), noting WPV increasing in Asian countries and reducing in Western countries. | long waiting time, overcrowding, high patient costs and less staff or resources. Adverse outcomes such as death of relative or loved one. Infrastructure and service issues and managing high patient turnover. | Review of literature across a range of developed and developing countries. | Young male individuals with lower education status and higher social profile. | Physical injury with 62.3% of these victims taking leave from work. Post-traumatic distress,  mental exhaustion and emotional distress. | Impact of resource availability and patient/doctor relationship. | Staff education such de-escalation and training. |
| Liu et al, 2020^39^ | Cross-sectional survey | Status of psychological WPV from coworkers and identify risk factors. | 92.1% experienced WPV, only 30.1% reported. | Longer term of service, more senior professional titles more likely to experience WPV. | 26 hospitals in 13 cities in China. | Surgeons (31%), senior nurses (18.1%). | Decreased enthusiasm for work. | Intention to quit, loss of resources. | Effective team communication, interdisciplinary education. |
| McGuire et al, 2021^40^ | Cross-sectional survey | Reporting behaviors of WPV in emergency department. | 71.6% experienced WPV in past 6 months. Majority indicated never or rarely reporting. Beliefs WPV violence is part of the job, lack of understanding around reporting systems. | Not reported. | 20 emergency departments in mid-west U.S. | Not reported. | Not reported. | Not reported. | Health administrators to understand causes of underreporting, continued staff education and encouragement of reporting. |
| Arnetz et al, 2017^41^ | Randomized controlled intervention | To assess the effect of intervention on Type 2 WPV. | Incident rate ratios significantly reduced in intervention units 6 months post, however there were no significant differences beyond 12 months. Rates of violence related injuries were lower in the intervention group. | Not reported. | Multi-site hospital system in mid-west U.S. | Not reported. | Physical and psychological harm, job dissatisfaction. | Loss of resources. | Development of an action plans based on unit level violence data (for intervention group) and no data (for comparison group). Recommends better reporting and unit level input into WPV prevention. |
| Spelten et al, 2020^42^ | Systematic review | Assess effectiveness of WPV interventions. | Low quality evidence that interventions focusing on the patient in the pre-event and event phase of WPV reduced aggression. | Not reported. | 4 studies from U.S. and 3 from Australia. Primarily psychiatric and nursing home settings. | Not reported. | Not reported. | Not reported. | Need for more RCTs with a published protocol, studies should include the victim and other stakeholders, expand settings to less controlled environments, expand interventions to post-event phase, need for homogenous outcome measures. |
| McGuire et al, 2022^43^ | Case study | Comparison of perception of violence between ED staff and law enforcement officers (LEO). | Not reported. | Not reported. | ED setting for hypothetical scenarios. | Various common patient factors including dementia, intoxication, delirium. | Not reported. | Not reported. | Need to consider staff perceptions of what constitutes violence in specific healthcare settings. |
| Wiskow, 2003^44^ | Report | Comparison of guidelines for reducing WPV. | More commonality between guidelines than not, evaluation of guidelines is lacking. | N/A | UK, U.S., Sweden, Australia. | N/A | N/A | N/A | Common recommendations include preventative measures, reporting and record keeping, engineering solutions, changes to work procedures, training and staffing. |
| Nyberg et al, 2020^45^ | Systematic review | Association between WPV and HCW's health. | Statistically significant associations between WPV and physical and mental health. | Not reported. | Majority of studies from Nordic countries. | Not reported. | Associations between physical violence and poor mental health. Statistically significant associations between psychological violence and sickness absence. | Time off work. | Further research using objective outcomes, improved exposure measures and focus on gender based violence. |
| Shaik et al, 2020^46^ | Cross-sectional survey | Magnitude and causes of WPV. | 38.4% reported experiencing any type of violence in past 6 months. | Death of loved one, serious illness, delay in care and quality of care. | HCW in 4 large cities in Pakistan. | Not reported. | Increased stress and burnout. | Decreased job performance. | Training of HCW, more resources, zero-tolerance policies. |
| Dye et al, 2020^47^ | Cross-sectional survey | COVID-19 related stigma and bullying against HCWs. | Increased bullying related to COVID-19 and loss of respect in the community. | Fear, panic, misinformation, anger. | Convenience sample through internet and social media channels of individuals who worked in healthcare settings. | Not reported. | WPV increases stress and exacerbates psychological sequelae resulting from moral injuries. Discrimination and stigma increase risk for mental illness, including anxiety, depression, post-traumatic stress disorder (PTSD) and suicidality. | Not reported. | Destigmatize HCWs through engagement with community and involvement of non-healthcare stakeholders. |
| Gimenez et al, 2021^48^ | Systematic review | Factors leading to burnout among nurses and physicians and protective factors. | Significant correlation between burnout symptoms and physical WPV. | For burnout; organizational type and structure (level of support, management style, work hours). | Review of literature across multiple databases. | Mainly patients. | WPV leads to high levels of observed burnout, emotional exhaustion, lower feelings of personal accomplishment, and therefore more negative thoughts about their competency. | Not reported. | Zero-tolerance policies, educational resources for HCWs, support networks, responsibility of government agencies. |
| Fang et al, 2018^49^ | Cross-sectional survey | Association between WPV and depressive symptoms. | Not reported. | Attitudes of patients, perceived efficacy of treatment and lack of professional skills of staff. Less than 1 year experience and working alone. | Nurses and physicians (Otorhinolaryngology) in Northern China. | Not reported. | 57% of all respondents who had experienced WPV had depressive symptoms, 71% who had experienced physical WPV had depressive symptoms. | Poor productivity and work quality. | Zero-tolerance policies, enhancing communication skills. |
| Kaur et al, 2020^50^ | Cross-sectional survey | Asses WPV against doctors and the impact on patient care. | 78% experienced violence in previous 12 months. 100% had ever experienced WPV. | Actual or perceived non-improvement of patient condition (40%), perception of wrong treatment (37%) Cost of care and unrealistic patient demands also reported. Younger age, unmarried and male and working in urban areas more likely to experience WPV. | Doctors working across multiple regions of India. | Family members or relatives main perpetrators. | Loss of self-esteem & feeling of shame; 52.2%. Stress/depression/anxiety/ideas of persecution; 51.2%. Sense of defeat 41.7%. Change place of work; 12.4%. Increased aggressiveness towards patients; 34.8% | Avoidance/missing work & Loss of productivity and income; 27.9%. Decrease in surgical and emergency management, increase in investigations and referrals. | Increased reporting and surveillance. Education regarding communication, prevention and management. |
| Khan et al, 2021^51^ | Cross-sectional survey | Prevalence and associated factors of WPV. | 51% any violence, verbal (45%), physical (24%). | Communication failures, unreasonable patient expectations, Working in emergency department or hospital wards in large hospitals. Staff shortages, lack of facilities, workload. | HCWs in private and public practice in Peshawar, Pakistan. | Relatives and patients. | Around two-thirds of the participants experienced mental health consequences. | Not reported. | Improvement in healthcare services, public awareness, control of entry of public, better security. |
| Hokee et al, 2023^52^ | Protocol for scoping review | Consideration of evidence relating to WPV against paramedics. | Evidence to suggest that violence against paramedic personnel is increasing. | N/A | Pre-hospital environment attended by paramedics. | Most commonly patients and family members however can include bystanders and general public. | Stress, a decrease in job satisfaction, increased anxiety, feelings of fear, anger and guilt and lack of empathy towards patients. Chronic stress which has been associated with substance abuse, anxiety, depression, burn-­out and even suicide. | Worse patient outcomes. | N/A |
| Wang et al, 2020^53^ | Cross-sectional survey | Effective of WPV on mental health of HCW during COVID-19 | 20% reported experiencing WPV during 1 month period of the COVID-19 pandemic and those who did were more likely to experience mental health issues. | Not reported. | Chinese HCW in 31 provinces. | Not reported. | WPV was correlated with ele­vated mental health problems including depression, anxiety and stress symptoms. | Worse clinical decision making leading to poorer patient outcomes. | Policy and legislative action including zero-tolerance and greater security infrastructure. |
| Ghareeb et al, 2021^54^ | Cross-sectional survey | Frequency, characteristics, effects and contributing factors to WPV during the COVID-19 pandemic. | 66% exposed to WPV in past 6 months. Low levels of reporting. | Patient expectations, long wait times, workload. | Government hospital in Jordan. | Patients relatives most common. | Psychological effects included feeling afraid and worried about people, and worried about talking about the incidents. | WPV negatively affected their work quality and may lead to high medical errors and low job satisfaction. | Increase HCW confidence in reporting, better security, improved communication. |
| Tiesman et al, 2022^55^ | Cross-sectional survey | Prevalence of WPV and association with mental health symptoms during the COVID-19 pandemic. | Not reported. | Public perception/attitude toward HCWs, longer hours worked. | Various office based and clinical settings within Public Health organizations. | Not reported. | Experiencing any type or combination of workplace violence was significantly associated with an increased likelihood of reporting depression, anxiety, suicidal ideation. | Ability to confront future Public Health crises. Increased turnover, burnout, and decreased job satisfaction. | Ongoing training, workplace support, and increased communication after a workplace violence incident. Increase reporting including legislative measures. Increasing workplace communication after a WPV incident. |
| Estryn-Behar et al, 2008^56^ | Cross-sectional survey | Prevalence of violence in nursing. | 22% of respondents experienced violence frequently. | Quality of team work, uncertainty regarding treatment, time pressure, working night shift, workload. | Highest frequency in Emergency, Psychiatric and Geriatrics. | Not reported. | Burnout, intent to leave nursing and intent to change institution. | Loss of nursing resources. | Management strategies adopting a multidisciplinary approach, additional resources, education of students and junior staff. |
| Dyrbye et al, 2022^57^ | Cross-sectional survey | Examine the relationship between mistreatment and discrimination and burnout. | 29% experienced racist or sexist remarks, 20% experienced unwanted sexual advances, 22% experienced patients or family refusing care because of physicians personal attributes in past 12 months. 15% reported physical harm. | Female, refusal of care experienced more often by non-white physicians. | AMA registered physicians in the U.S. | Not reported. | Depressive symptoms, burnout, job dissatisfaction, and turnover, emotional exhaustion. | Increase in burnout and depersonalization is associated with higher odds of a physician believing they have recently committed a major medical error. Time away from work. | Promotion of an environment of safety, equality and inclusion. Coordinated strategy involving multiple stakeholders. |
| Sun et al, 2017^58^ | Cross-sectional survey | Incidence of WPV, association between exposure to WPV and health outcomes of Doctors. | 83.4% of participants reported having experienced one or more types of WPV during the last 12 months. Verbal abuse 76.2%, made difficulties 58.3%, smear reputation 40.8%, mobbing behavior 40.2%, intimidation behavior 27.6%, physical violence 24.1% and sexual harassment 7.8% | Doctor-patient relationship, concentration of high quality resources in major cities resulting in strain on these hospitals, weak primary health-care system, high costs of healthcare. | Multiple large hospitals in China. | Not reported. | Psychological stress, poor sleep quality, decline in job performance, increased turnover intentions and reduced job satisfaction. | Not reported. | Not reported. |
| Zhang et al, 2018^59^ | Cross-sectional survey | Describe the current state of workplace violence (WPV) and compassionate behavior towards nurses and to explain how they affect nurses’ health. | 75% experienced WPV in previous 12 months. | Fatigue, stress and work dissatisfaction. | Nurses working across 8 provinces in China. | Patients and family members. | Psychological stress, physical health impacts, sleep quality and subjective health status. | Fatigue due to sleep quality impacts increasing risk of medical errors. | Compassionate behavior from colleagues was beneficial to nurses health outcomes. |
| Havaei et al, 2020^60^ | Cross-sectional survey | Role of the work environment in moderating the mediating effect WPV has on health outcomes. | On average nurses were exposed to WPV a few times per year. | Not reported. | Surgical nurses in British Columbia, Canada. | Not reported. | Burnout mediated the relationship between workplace violence and health outcomes; musculoskeletal injuries, anxiety disorders and sleep disturbances. WPV was most strongly associated with negative psychological outcomes, including post-traumatic stress disorder, depression, anxiety, sleep disturbances and fatigue. Healthier work environments had higher reports of psychological and physical harm. | Not reported. | Not reported. |
| Jakobsson et al, 2020^61^ | Cross-sectional survey | Experiences and approaches to management of WPV on surgical wards. | Not reported. | Illness related, substance addiction and withdrawal. | Surgical ward in a metropolitan hospital in Sweden. | Dementia, delirium. | Feeling exposed, scared, and unprotected.  Stress, insecurity, anxiety, and fear negatively affected their wellbeing. Physical symptoms such as headaches, stomach problems, sleeping disorders, loss of appetite, and difficulties in concentrating. Negative effect on the healthcare professionals’ level of skill and efficiency at work. | Increases the risk for ill-health, high staff turnover, and a deterioration in the quality of care. | HCWs employed ad-hoc strategies based on experience and support from colleagues. Despite voluntary online training being available HCWs felt unequipped to deal with WPV. Recommendations to develop specific guidelines and education material for HCWs. |
| Dagnaw et al, 2021^62^ | Cross-sectional survey | Magnitude and causes of WPV. | 44.5% of HCW experienced WPV. | Perceived quality of care, long wait times, miscommunication. Working in a labor ward, female, less than 5 years experience and low number of staff. | Obstetrics and gynecology department in a referral hospital in Ethiopia. | Patients most commonly. | Stress, physical morbidity, burn out, poor job satisfaction, high turnover. | Negligent of health care providers, ineffective clinical performance, compromised quality health care. | Incident resolution protocol, legislative measures to increase reporting and more severe penalties for perpetrators. |
| Alhamad et al, 2021^63^ | Cross-sectional survey | Evaluate properties of WPV including type, characteristics of perpetrators, timing and impacts. | 63.1% in previous 12 months. | Male gender. | Doctors working in public, private and military hospitals in Jordan. | Mainly relatives followed by patients. | Negative impacts on their physical and mental health, dissatisfaction with management handling of violence. | 71.8% reported that their job performance was affected, and several work changes were related to violence incidents. | Awareness campaigns and staff training. |
| Guo et al, 2022^64^ | Systematic review | Summarize the current evidence of the impact of unacceptable behavior occurring between HCWs on clinical performance and patient outcomes. | Overall evidence suggests WPV negatively impacts quality of care, workplace productivity and patient outcomes. | Not reported. | 36 studies relating to impacts of WPV on HCWs. Mostly from North America, however also from Europe, Middle East, Asia and Oceania. | Not reported. | Reduced staff performance. | Impacts of performance, quality of care, reduced productivity and worse patient outcomes. | Target future research at evaluating interventions that may reduce the frequency and impact of unacceptable behavior. |
| Chakraborty et al, 2022^65^ | Systematic review and meta-analysis | Exploring violence against physicians and nurses. | A majority of respondents in included studies indicated they were exposed to WPV during the previous 12 months. Under-reporting common. | Excessive wait times most common, also lack of communication and unrealistic expectations. Working in emergency departments and ICU, female. | Multiple databases searched for articles relating to violence or aggression against physicians and nurses. | Patients and relatives. | Lack of job satisfaction, intention to leave job, psychological stress, sleep quality and health, worker–patient relationship. | Quality of patient care. | Strengthening security, improving communication and public awareness. |
| WHO, 2016^66^ | Report | Global strategy to support objective of universal health coverage. | Identified significant shortage of HCWs required to meet demand in 2030. | N/A | Global healthcare focus. | N/A | N/A | N/A | Range of policy options for all stakeholders to aid in the necessary increase in HCWs. |
| Farrell et al, 2014^67^ | Cross-sectional survey | Experiences of WPV, the presence of protective factors and their effectiveness. | Not reported. | Patient factors including illness and disagreement with treatment. Built environment factors and working conditions. | Multiple nursing settings. | Not reported. | Feeling angry, emotionally upset, more fearful about work-related violence, and feel less safe time-off, or leaving their job. | Loss of resources. | Most important protective factors identified include appropriate physical environment, surveillance, means of safe retreat. |
| Bryant-Genevier et al, 2021^68^ | Cross-sectional survey | Evaluate mental health conditions amongst public health workers in the U.S. during COVID-19. | Not reported. | Not reported. | Public health workers, most likely non-clinical settings. | Not reported. | Mental health conditions; 52.8%. depression (30.8%), anxiety (30.3%), PTSD (36.8%), or suicidal ideation (8.4%). | Absenteeism, high turnover, lower productivity, and lower morale. | Proposed expanding staffing size and more flexible work hours. |
| CDC, 2006^69^ | Report | Expert collaboration to identify and implement effective prevention programs and strategies, identify barriers to prevention, identify current research and communication needs, and the advancement of research and prevention through effective partnerships. | Gaps include increasing evaluation research, improving reporting, data sharing, cooperation between employers and HCWs. | N/A | U.S. healthcare system. | N/A | N/A | Increased costs and loss of resources. | Range of policy options and priority research areas. |
| Wassell, 2009^70^ | Systematic review | Effectiveness of interventions. | Significant variability of interventions with low evidence of improvements for some. | Not reported. | Multiple nursing settings. | Not reported. | Mental and physical health impacts. | Not reported. | Significant variability in the efficacy of training interventions. |
| Kumari et al, 2022^71^ | Systematic review | Synthesize evidence on intervention strategies for WPV. | Educational interventions showed effect in improving HCW's perceived ability to deal with situations that lead to WPV. | Not reported. | 17 studies (from North America, Asia, Africa, Middle East, Australia, New Zealand and Europe) identified through multiple databases. | Not reported. | Interventions may reduce mental health impacts and increase performance. | Not reported. | Improve HCW communication skills, better standardization of research processes to identify efficacy of interventions. |
| Baby et al, 2019^72^ | Randomized controlled intervention | Efficacy of communication skills and mindfulness training in reducing perception of WPV. | Reduction in perceived patient aggression at 3 and 6 months post. | Not reported. | Healthcare support workers in New Zealand. | Most commonly family members. | Not reported. | Not reported. | Improving HCW communication skills. |
| Larson et al, 2019^73^ | Non-randomized intervention study | Effectiveness of a communication intervention involving a violent patient handover between colleagues. | Improvement in feelings of safety and improvement in satisfaction with handover process. | Not reported. | Large tertiary hospital in mid-west U.S. | Not reported. | Not reported. | Not reported. | Better communication tools for handover of potentially violent patients may help to reduce the number and severity of violent incidents. |
| Hardy et al, 2017^74^ | Non-randomized intervention study | Feasibility of using body worn cameras in an inpatient mental health setting. | Wearing of body worn cameras is feasible however shows mixed results in effect on WPV. | Not reported. | Single psychiatric unit in England. | Not reported. | Staff viewed the use of body worn cameras as beneficial. | Not reported. | Further research required to understand the impact of body worn cameras on WPV. |
| Quigley et al, 2020^75^ | Descriptive study | Types of patients' WPV against nurses as observed through video surveillance and interventions initiated by video technicians. | 3% of patients observed were identified as being at risk of aggressive/violent behavior with only 0.6% of those patients demonstrating such behavior. Only 15% of patients who exhibited aggressive/violent behavior were identified. | Not reported. | 73 Hospitals in the U.S. | Majority male (59%) | Video surveillance may help to prevent violent incidence. | Not reported. | Supports the use of video surveillance to help identify and prevent/mitigate WPV. Training programs required along with organizational leadership to support effective implementation. |
| National Nurses United, 2022^76^ | Press release | Support for legislation to prevent WPV in health sector. | N/A | N/A | U.S. healthcare system. | N/A | Mental and physical health impacts. | N/A | Obligations on healthcare employers to develop WPV prevention plans. |
| Casteel et al, 2009^77^ | Pre-post intervention study | Changes in violent event rates after legislative intervention requiring hospitals to implement a comprehensive security plan. | Assault rates showed a modest decrease post intervention. Reducing from 0.68 to 0.6 per 100,000 employee hours. | Not reported. | Hospitals in California and New Jersey. | Not reported. | Reduced assault rates. | Not reported. | Policy may be effective measure to aid in reduction of WPV. |
| Odes et al, 2022^78^ | Mixed methods | Impact of legislated WPV reporting requirements. | Wide range of approaches to reporting across different hospitals. | Not reported. | California healthcare system. | Not reported. | Not reported. | Not reported. | Prioritization of complete and accurate data collection. |
| OSHA, 2016^79^ | Guideline | Guide to aid employers reduce risks of WPV. | 70-74% of all workplace assaults in U.S. between 2011 and 2013 occurred in the health and social services sector. | Inpatient and acute care, psychiatric and geriatric environments most at risk. Lack of policies and staff training, lack of resources, high staff turnover and lack of support from management. | U.S. healthcare system. | N/A | Mental and physical health impacts. | Time off work, loss of resources. | Written WPV prevention program including management responsibility, worksite analysis, hazard prevention and control, training, reporting and record keeping. |
| Arnetz, 2022^80^ | Commentary | Expert review and comment on WPV prevention standards. | Underreporting a significant barrier to effective intervention, new accreditation standards an important step toward awareness, prevention and management of WPV. | N/A | U.S. healthcare system. | N/A | N/A | N/A | New accreditation guidelines require annual worksite analysis and management of risks, continuous data monitoring and reporting of WPV risks, staff education and training, WPV prevention programs. |
| Barach et al, 2000^81^ | Clinical Review | Review of non-medical near miss reporting systems. | Application of lessons from non-medical industries on near miss reporting systems. | N/A | Multiple high-risk, non-medical, reporting systems. | N/A | N/A | N/A | N/A |

**References**

1 American College of Emergency Physicians. Poll: ED Violence is on the Rise [Internet]. Irving (TX): American College of Emergency Physicians; 2022 1 Aug [cited 2023 Nov 25]. Available from: <https://www.emergencyphysicians.org/article/er101/poll-ed-violence-is-on-the-rise>

2 Massachusetts Health and Hospital Association. Workplace violence at Massachusetts healthcare facilities: An untenable situation and a call to protect the workforce. [Internet]. Burlington (MA): Massachusetts Health and Hospital Association; 2023 Jan [cited 2023 Nov 25]. Available from: <https://www.mhalink.org/reportsresources/workplaceviolencereport/>

3 Gamble M. Senators intro bill to make assault of hospital workers a federal crime. Becker’s Hospital Review [Internet]. 2023 Sep 13 [cited 2023 Nov 25]: Legal and Regulatory Issues. Available from: <https://www.beckershospitalreview.com/legal-regulatory-issues/senators-intro-bill-to-make-assault-of-hospital-workers-a-federal-crime.html>

4 U.S. Bureau of Labor Statistics. Injuries, Illness and Fatalities: Factsheet, Workplace Violence in Healthcare [Internet]. Washington DC: U.S. Bureau of Labor Statistics; 2018 [cited 2023 Feb 2]. Available from: <https://www.bls.gov/iif/factsheets/workplace-violence-healthcare-2018.htm#:~:text=%E2%80%8B%20Source%3A%20U.S.%20Bureau%20of%20Labor%20Statistics.,-View%20data&text=In%202018%2C%20workplace%20homicides%20in,5%2C250%20workplace%20fatalities%20in%202018>

5 Eala M, Maslog E, Robredo J, Pedro R, Medina P, De la Paz E, Lasco G. Violence against health-care workers in the Philippines. *Lancet* [Internet]. 2022 May 28 [cited 2023 Feb 3];**399(10340):**2012–2013. Available from <https://www.thelancet.com/journals/lancet/article/PIIS0140-6736(22)00785-1/fulltext>. doi: 10.1016/S0140-6736(22)00785-1

6 Lorettu L, Nivoli A, Daga I, Milia P, Depalmas C, Nivoli G, Bellizzi S. Six things to know about the homicides of doctors: a review of 30 years from Italy. *BMC Public Health* [Internet]. 2021 Jul 5 [cited 2023 Feb 27];**21:**1318. Available from: <https://bmcpublichealth.biomedcentral.com/articles/10.1186/s12889-021-11404-5>. doi: 10.1186/s12889-021-11404-5

7 Kelly N. ‘Global crisis’ of violence: 161 healthcare workers were killed last year, study finds. *The Guardian* [Internet]. 2022 May 25 [cited 2023 Feb 12]. Available from: <https://www.theguardian.com/global-development/2022/may/24/global-crisis-of-violence-161-healthcare-workers-were-killed-last-year-study-finds>

8 Pan Y, Yang X, He J, Yan H, Zhan X, Hui F, Qiao Q, Zhou D, Jin H. To be or not to be a doctor, that is the question: a review of serious incidents of violence against doctors in China from 2003–2013. *J Public Health* [Internet]. 2015 Mar 1 [cited 2023 Feb 18];**23:**111–116. Available from: <https://link.springer.com/article/10.1007/s10389-015-0658-7>. doi: [10.1007/s10389-015-0658-7](https://doi.org/10.1007/s10389-015-0658-7)

9 Higgins JPT, Thomas J, Chandler J, Cumpston M, Li T, Page MJ, Welch VA (editors). Cochrane Handbook for Systematic Reviews of Interventions version 6.4 (updated August 2023). Cochrane, 2023. Available from [www.training.cochrane.org/handbook](http://www.training.cochrane.org/handbook)

10 Moola S, Munn Z, Tufanaru C, Aromataris E, Sears K, Sfetcu R, Currie M, Qureshi R, Mattis P, Lisy K, Mu P-F. Chapter 7: Systematic reviews of etiology and risk . In: Aromataris E, Munn Z (Editors). JBI Manual for Evidence Synthesis. JBI, 2020. Available from <https://synthesismanual.jbi.global>

11 Aromataris E, Fernandez R, Godfrey C, Holly C, Kahlil H, Tungpunkom P. Summarizing systematic reviews: methodological development, conduct and reporting of an Umbrella review approach. Int J Evid Based Healthc. 2015;13(3):132-40

12 McArthur A, Klugarova J, Yan H, Florescu S. Innovations in the systematic review of text and opinion. Int J Evid Based Healthc. 2015;13(3):188–195

13 Glenton C, Bohren MA, Downe S, Paulsen EJ, Lewin S. Cochrane Qualitative Evidence Synthesis: Protocol and review template. Version 1.4b. Cochrane Person Centred Care, Health Systems and Public Health and Cochrane Norway; 2023. Available at: <https://doi.org/10.5281/zenodo.5101812>

14 Lewin S, Booth A, Glenton C, Munthe-Kaas H, Rashidian A, Wainwright M, Bohren MA, Tunçalp Ö, Colvin CJ, Garside R, Carlsen B, Langlois EV, Noyes J. Applying GRADE-CERQual to qualitative evidence synthesis findings: introduction to the series. Implement Sci. 2018 Jan 25;13(Suppl 1):2. doi: 10.1186/s13012-017-0688-3

15 Kumar M, Verma M, Das T, Pardeshi G, Kishore J, Padmanandan A. A study of workplace violence experienced by doctors and associated risk factors in a tertiary care hospital of South Delhi, India. *J Clin Diagn Res* [Internet]. 2016 Nov 1 [cited 2023 Feb 7];**10(11):**LC06–LC10. Available from: <https://www.ncbi.nlm.nih.gov/pmc/articles/PMC5198359/#:~:text=Conclusion,of%20such%20cases%20were%20reported>. doi: 10.7860/JCDR/2016/22306.8895

16 Duan X, Ni X, Shi L, et al. The impact of workplace violence on job satisfaction, job burnout, and turnover intention: the mediating role of social support. *Health Qual Life Outcomes.* 2019 May 30 [cited 2023 Feb 21];**17(1):**93. Available from: <https://hqlo.biomedcentral.com/articles/10.1186/s12955-019-1164-3>. doi: 10.1186/s12955-019-1164-3

17 Nowrouzi-Kia B, Chai E, Usuba K, Nowrouzi-Kia B, Casole J. Prevalence of type II and type III workplace violence against physicians: a systematic review and meta-analysis. *Int J Occup Environ Med* [Internet]*.* 2019 Jul 1 [cited 2023 Feb 16];**10(3):**99–110. Available from: <https://www.ncbi.nlm.nih.gov/pmc/articles/PMC6708400/>. doi: 10.15171/ijoem.2019.1573

18 Byon H, Sagherian K, Kim Y, Lipscomb J, Crandall M, Steege L. Nurses’ experience with type II workplace violence and underreporting during the COVID-19 pandemic. *Workplace Health Saf* [Internet]. 2021 Aug 3 [cited 2023 Mar 14];**3:**21650799211031233. Available from: <https://journals.sagepub.com/doi/10.1177/21650799211031233?url_ver=Z39.88-2003&rfr_id=ori:rid:crossref.org&rfr_dat=cr_pub%20%200pubmed>. doi: 10.1177/21650799211031233

19 Aljohani B, Burkholder J, Tran Q, Chen C, Beisenova K, Pourmand A. Workplace violence in the emergency department: a systematic review and meta-analysis. *Public Health* [Internet]. 2021 Jul 8 [cited 2023 Mar 15];**196:**186–197. Available from: <https://www.sciencedirect.com/science/article/pii/S0033350621000676>. doi: 10.1016/j.puhe.2021.02.009

20 Civilotti C, Berlanda S, Iozzino L. Hospital-based healthcare workers victims of workplace violence in Italy: a scoping review. *Int J Environ Res Public Health* [Internet]*.* 2021 May 29 [cited 2023 Feb 18];**18(11):**5860. Available from: <https://www.mdpi.com/1660-4601/18/11/5860>. doi: 10.3390/ijerph18115860

21 Spelten E, van Vuuren J, O'Meara P, et al. Workplace violence against emergency health care workers: what strategies do workers use? *BMC Emerg Med* [Internet]*.* 2022 May 6 [cited 2023 Feb 16];**22(1):**78. Available from: <https://bmcemergmed.biomedcentral.com/articles/10.1186/s12873-022-00621-9>. doi: 10.1186/s12873-022-00621-9

22 Ramzi Z, Fatah P, Dalvandi A. Prevalence of workplace violence against healthcare workers during the COVID-19 pandemic: a systematic review and meta-analysis. *Front Psychol* [Internet]*.* 2022 May 30 [cited 2023 Feb 14];**13:**896156. Available from: <https://www.ncbi.nlm.nih.gov/pmc/articles/PMC9195416/>. doi: 10.3389/fpsyg.2022.896156

23 Lei Z, Yan S, Jiang H, et al. Prevalence and risk factors of workplace violence against emergency department nurses in China. *Int J Public Health* [Internet]. 2022 Aug 4 [cited 2023 Feb 20];**67:**1604912. Available from: <https://www.ssph-journal.org/articles/10.3389/ijph.2022.1604912/full>. doi: 10.3389/ijph.2022.1604912

24 El-Zoghby S, Ibrahim M, Zaghloul N, Shehata S, Farghaly R. Impact of workplace violence on anxiety and sleep disturbances among Egyptian medical residents: a cross-sectional study. *Hum Resour Health* [Internet]*.* 2022 Dec 19 [cited 2023 Feb 18];**20(1):**84. Available from: <https://human-resources-health.biomedcentral.com/articles/10.1186/s12960-022-00786-1>. doi: 10.1186/s12960-022-00786-1

25 Vento S, Cainelli F, Vallone A. Violence against healthcare workers: a worldwide phenomenon with serious consequences. *Front Public Health* [Internet]*.* 2020 Sep 18 [cited 2023 Jan 28];**8:**570459. Available from: <https://www.ncbi.nlm.nih.gov/pmc/articles/PMC7531183/>. doi: 10.3389/fpubh.2020.570459

26 Caruso R, Toffanin T, Folesani F, et al. Violence against physicians in the workplace: trends, causes, consequences, and strategies for intervention. *Curr Psychiatry Rep* [Internet]. 2022 Nov 9 [cited 2023 Feb 6];**12:**911–924. Available from: <https://link.springer.com/article/10.1007/s11920-022-01398-1>. doi: 10.1007/s11920-022-01398-1

27 Iacobucci G. Violent incidents at GP surgeries double in five years, BMJ investigation finds. *BMJ* [Internet]. 2022 May 31 [cited 2023 Mar 25];**377:**o1333. Available from: <https://www.bmj.com/content/bmj/377/bmj.o1333.full.pdf>. doi: 10.1136/bmj.o1333

28 Dopelt K, Davidovitch N, Stupak A, Ben Ayun R, Lev Eltsufin A, Levy C. Workplace violence against hospital workers during the COVID-19 pandemic in Israel: implications for public health. *Int J Environ Res Public Health* [Internet]. 2022 Apr 12 [cited 2023 Mar 4];**19(8):**4659. Available from: <https://www.mdpi.com/1660-4601/19/8/4659>. doi: 10.3390/ijerph19084659

29 Viottini E, Politano G, Fornero G, et al. Determinants of aggression against all health care workers in a large-sized university hospital. *BMC Health Serv Res* [Internet]. 2020 Mar 16 [cited 2023 Feb 27];**1:**215. Available from: <https://bmchealthservres.biomedcentral.com/articles/10.1186/s12913-020-05084-x#citeas>. doi: 10.1186/s12913-020-05084-x

30 Liu J, Zheng J, Liu K, Liu X, Wu Y, Wang J, You L. Workplace violence against nurses, job satisfaction, burnout, and patient safety in Chinese hospitals. *Nurs Outlook* [Internet]. 2019 May 2 [cited 2023 Mar 12];**67(5):**558–566. Available from: <https://www.sciencedirect.com/science/article/pii/S0029655418306523>. doi: 10.1016/j.outlook.2019.04.006

31 Tian K, Xiao X, Zeng R, et al. Prevalence of workplace violence against general practitioners: a systematic review and meta-analysis. *Int J Health Plann Manage* [Internet]*.* 2021 Dec 17 [cited 2023 Mar 2];**37(3):**1238-1251. Available from: <https://onlinelibrary.wiley.com/doi/abs/10.1002/hpm.3404>. doi: 10.1002/hpm.3404

32 Choi S, Lee H. Workplace violence against nurses in Korea and its impact on professional quality of life and turnover intention. *J Nurs Manag* [Internet]*.* 2017 May 25 [cited 2023 Jan 24];**25(7):**508–518. Available from: <https://onlinelibrary.wiley.com/doi/10.1111/jonm.12488>. doi: 10.1111/jonm.12488

33 Grossman D, Choucair B. Violence and the US health care sector: burden and response. *Health Aff (Millwood)* [Internet]*.* 2019 Oct [cited 2023 Mar 24];**38(10):**1638–1645. Available from: <https://www.healthaffairs.org/doi/10.1377/hlthaff.2019.00642>. doi: 10.1377/hlthaff.2019.00642

34 Reed T, Millman J. Hospitals and clinics are now among America's most dangerous workplaces. Axios [Internet]. 2023 Aug 10 [cited 2023 Aug 16]. Available from: [https://www.axios.com/2023/08/10/escalating-violence-americas-hospitals#](https://www.axios.com/2023/08/10/escalating-violence-americas-hospitals)

35 Massaro M, Tamburro P, La Torre M, et al. Non-pharmaceutical interventions and the infodemic on Twitter: lessons learned from Italy during the Covid-19 pandemic. *J Med Syst* [Internet]*.* 2021 Mar 6 [cited 2023 May 19];**45(4):**50. Available from: <https://pubmed.ncbi.nlm.nih.gov/33675427/>. doi: 10.1007/s10916-021-01726-7

36 Brigo F, Zaboli A, Rella E, et al. The impact of COVID-19 pandemic on temporal trends of workplace violence against healthcare workers in the emergency department. *Health Policy* [Internet]. 2022 Sep 23 [cited 2023 Apr 2];**126(11):**1110–1116. Available from: <https://www.sciencedirect.com/science/article/pii/S0168851022002536>. doi: 10.1016/j.healthpol.2022.09.010

37 Phillips J. Workplace Violence against Health Care Workers in the United States. *N Engl J Med* [Internet]*.* 2016 April 28 [cited 2023 Jan 27];**374(17):**1661–9. Available from: <https://www.nejm.org/doi/10.1056/NEJMra1501998?url_ver=Z39.88-2003&rfr_id=ori:rid:crossref.org&rfr_dat=cr_pub%20%200pubmed>. doi: 10.1056/NEJMra1501998

38 Kumari A, Kaur T, Ranjan P, Chopra S, Sarkar S, Baitha U. Workplace violence against doctors: characteristics, risk factors, and mitigation strategies. *J Postgrad Med* [Internet]. 2020 Jul [cited 2023 Mar 4];**66(3):**149–154. Available from: <https://pubmed.ncbi.nlm.nih.gov/32675451/>. doi: 10.4103/jpgm.JPGM_96_20

39 Liu X, Wang L, Chen W, Wu X. A cross-sectional survey on workplace psychological violence among operating room nurses in Mainland China. *Appl Nurs Res* [Internet]*.* 2020 Aug 25 [cited 2023 Apr 14];**57:**151349. Available from: <https://europepmc.org/article/med/32893086>. doi: 10.1016/j.apnr.2020.151349

40 McGuire S, Finley J, Gazley B, Mullan A, Clements C. 18 workplace violence reporting behaviors in emergency departments across a health system. *Annals of Emergency Medicine* [Internet]. 2021 Oct [cited 2023 Mar 16];**78(4):**8. Available from: <https://www.annemergmed.com/article/S0196-0644(21)00867-2/fulltext>. doi: <https://doi.org/10.1016/j.annemergmed.2021.09.026>

41 Arnetz J, Hamblin L, Russell J, et al. Preventing patient-to-worker violence in hospitals: outcome of a randomized controlled intervention. *J Occup Environ Med* [Internet]. 2017 Jan [cited 2023 May 27];**59(1):**18–27. Available from: <https://journals.lww.com/joem/abstract/2017/01000/preventing_patient_to_worker_violence_in.4.aspx>. doi: 10.1097/JOM.0000000000000909

42 Spelten E, Thomas B, O'Meara PF, Maguire B, FitzGerald D, Begg S. Organisational interventions for preventing and minimising aggression directed towards healthcare workers by patients and patient advocates. *Cochrane Database Syst Rev* [Internet]. 2020 Apr 29 [cited 2023 Apr 4];**4(4):**CD012662. Available from: <https://www.cochranelibrary.com/cdsr/doi/10.1002/14651858.CD012662.pub2/full>. doi: 10.1002/14651858.CD012662.pub2

43 McGuire S, Mullan A, Clements C. Workplace violence in the emergency department: case study on staff and law enforcement disagreement on reportable crimes. *Int J Environ Res Public Health* [Internet]*.* 2022 Jun 2 [cited 2023 May 29];**19(11):**6818. Available from: <https://www.mdpi.com/1660-4601/19/11/6818>. doi: 10.3390/ijerph19116818

44 Wiskow C. Guidelines on workplace violence in the health sector: Comparison of major known national guidelines and strategies: United Kingdom, Australia, Sweden, USA. Geneva: World Health Organization; 2003. 37 p.

45 Nyberg A, Kecklund G, Hanson L, Rajaleid K. Workplace violence and health in human service industries: a systematic review of prospective and longitudinal studies. *Occup Environ Med* [Internet]*.* 2020 May 15 [cited 2023 Apr 21];**78(2):**69–81. Available from: <https://oem.bmj.com/content/78/2/69>. doi: 10.1136/oemed-2020-106450

46 Shaikh S, Baig L, Hashmi I, et al. The magnitude and determinants of violence against healthcare workers in Pakistan. *BMJ Glob Health* [Internet]. 2020 Apr 15 [cited 2023 Feb 18];**5(4):**e002112. Available from: <https://gh.bmj.com/content/5/4/e002112>. doi: 10.1136/bmjgh-2019-002112

47 Dye T, Alcantara L, Siddiqi S, et al. Risk of COVID-19-related bullying, harassment and stigma among healthcare workers: an analytical cross-sectional global study. *BMJ Open* [Internet]. 2020 Dec 30 [cited 2023 Mar 2];**10(12):**e046620. Available from: <https://bmjopen.bmj.com/content/10/12/e046620.share>. doi: 10.1136/bmjopen-2020-046620

48 Giménez Lozano J, Martínez Ramón J, Morales Rodríguez F. Doctors and Nurses: a systematic review of the risk and protective factors in workplace violence and burnout. *Int J Environ Res Public Health* [Internet]*.* 2021 Mar 22 [cited 2023 Feb 16];**18(6):**3280. Available from: <https://www.mdpi.com/1660-4601/18/6/3280>. doi: 10.3390/ijerph18063280

49 Fang H, Zhao X, Yang H, et al. Depressive symptoms and workplace-violence-related risk factors among otorhinolaryngology nurses and physicians in Northern China: a cross-sectional study. *BMJ Open* [Internet] 2018 Jan 27 [cited 2023 Feb 23];**8(1):**e019514. Available from: <https://bmjopen.bmj.com/content/8/1/e019514>. doi: 10.1136/bmjopen-2017-019514#44

50 Kaur A, Ahamed F, Sengupta P, Majhi J, Ghosh T. Pattern of workplace violence against doctors practising modern medicine and the subsequent impact on patient care, in India. *PLoS One* [Internet]. 2020 Sep 18 [cited 2023 Feb 3];**15(9):**e0239193. Available from: <https://journals.plos.org/plosone/article?id=10.1371/journal.pone.0239193>. doi: 10.1371/journal.pone.0239193

51 Khan M, Haq Z, Khan M, et al. Prevalence and determinants of violence against health care in the metropolitan city of Peshawar: a cross sectional study. *BMC Public Health* [Internet]. 2021 Feb 10 [cited 2023 Apr 28];**21(1):**330. Available from: <https://bmcpublichealth.biomedcentral.com/articles/10.1186/s12889-021-10243-8>. doi: 10.1186/s12889-021-10243-8

52 Hokee M, Makkink A, Vincent-Lambert C. Workplace violence against paramedic personnel: a protocol for a scoping review. *BMJ Open* [Internet]. 2023 Jan 13 [cited 2023 Feb 18];**13:**e067246. Available from: <https://bmjopen.bmj.com/content/13/1/e067246>. doi:10.1136/bmjopen-2022-067246

53 Wang W, Lu L, Kelifa M, et al. Mental health problems in Chinese healthcare workers exposed to workplace violence during the COVID-19 outbreak: a cross-sectional study using propensity score matching analysis. *Risk Manag Healthc Policy* [Internet]*.* 2020 Dec 3 [cited 2023 May 4];**13:**2827–2833. Available from: <https://www.tandfonline.com/doi/full/10.2147/RMHP.S279170>. doi: 10.2147/RMHP.S279170

54 Ghareeb N, El-Shafei D, Eladl A. Workplace violence among healthcare workers during COVID-19 pandemic in a Jordanian governmental hospital: the tip of the iceberg. *Environ Sci Pollut Res Int* [Internet]*.* 2021 Jun 26 [cited 2023 May 2];**28(43):**61441–61449. Available from: <https://link.springer.com/article/10.1007/s11356-021-15112-w>. doi: 10.1007/s11356-021-15112-w

55 Tiesman H, Hendricks S, Wiegand D, et al. Workplace violence and the mental health of public health workers during COVID-19. *Am J Prev Med* [Internet]. 2022 Nov 19 [cited 2023 Jun 4];**64(3):**315–325. Available from: <https://www.ajpmonline.org/article/S0749-3797(22)00507-4/pdf>. doi: 10.1016/j.amepre.2022.10.004

56 Estryn-Behar M, van der Heijden B, Camerino D, et al. Violence risks in nursing-results from the European 'NEXT' Study. *Occup Med* [Internet]. 2008 Jan 21 [cited 2023 Feb 22];**58(2):**107–14. Available from: <https://academic.oup.com/occmed/article/58/2/107/1389848>. doi: 10.1093/occmed/kqm142

57 Dyrbye L, West C, Sinsky C, et al. Physicians' experiences with mistreatment and discrimination by patients, families, and visitors and association with burnout. *JAMA Netw Open* [Internet]*.* 2022 May 19 [cited 2023 Feb 21];**5(5):**e2213080. Available from: <https://jamanetwork.com/journals/jamanetworkopen/fullarticle/2792386>. doi: 10.1001/jamanetworkopen.2022.13080

58 Sun T, Gao L, Li F, et al. Workplace violence, psychological stress, sleep quality and subjective health in Chinese doctors: a large cross-sectional study. *BMJ Open* [Internet]. 2017 Dec 7 [cited 2023 Mar 17];**7(12):**e017182. Available from: <https://bmjopen.bmj.com/content/7/12/e017182.abstract>. doi: 10.1136/bmjopen-2017-017182

59 Zhang S, Liu W, Wang J, et al. Impact of workplace violence and compassionate behaviour in hospitals on stress, sleep quality and subjective health status among Chinese nurses: a cross-sectional survey. *BMJ Open* [Internet]. 2018 Oct 3 [cited 2023 May 6];**8(10):**e019373. Available from: <https://bmjopen.bmj.com/content/8/10/e019373.info>. doi: 10.1136/bmjopen-2017-019373

60 Havaei F, Astivia O, MacPhee M. The impact of workplace violence on medical-surgical nurses' health outcome: a moderated mediation model of work environment conditions and burnout using secondary data. *Int J Nurs Stud* [Internet]*.* 2020 Sep [cited 2023 May 2];**109:**103666. Available from: <https://www.sciencedirect.com/science/article/pii/S0020748920301504?via%3Dihub>. doi: 10.1016/j.ijnurstu.2020.103666

61 Jakobsson J, Axelsson M, Örmon K. The face of workplace violence: experiences of healthcare professionals in surgical hospital wards. *Nurs Res Pract* [Internet]*.* 2020 May 28 [cited 2023 Apr 21];**2020:**1854387. Available from <https://www.hindawi.com/journals/nrp/2020/1854387/>. doi: 10.1155/2020/1854387

62 Dagnaw E, Bayabil A, Yimer T, Nigussie T. Working in labor and delivery unit increases the odds of work place violence in Amhara region referral hospitals: cross-sectional study. *PLoS One* [Internet]. 2021 Oct 20 [cited 2023 Feb 4];**16(10):**e0254962. Available from: <https://journals.plos.org/plosone/article?id=10.1371/journal.pone.0254962> doi: 10.1371/journal.pone.0254962

63 Alhamad R, Suleiman A, Bsisu I, et al. Violence against physicians in Jordan: An analytical cross-sectional study. *PLoS One* [Internet]. 2021 Jan 25 [cited 2023 Mar 28];**16(1):**e0245192. Available from: <https://journals.plos.org/plosone/article?id=10.1371/journal.pone.0245192>. doi: 10.1371/journal.pone.0245192

64 Guo L, Ryan B, Leditschke I, et al. Impact of unacceptable behaviour between healthcare workers on clinical performance and patient outcomes: a systematic review. *BMJ Qual Saf* [Internet]*.* 2022 Jan 19 [cited 2023 Feb 3];**31(9):**679–687. Available from: <https://qualitysafety.bmj.com/content/31/9/679>. doi: 10.1136/bmjqs-2021-013955

65 Chakraborty S, Mashreky S, Dalal K. Violence against physicians and nurses: a systematic literature review. *Z Gesundh Wiss* [Internet]. 2022 Jan 22 [cited 2023 Apr 18];**30(8):**1837–1855. Available from: <https://link.springer.com/article/10.1007/s10389-021-01689-6>. doi: 10.1007/s10389-021-01689-6

66 World Health Organization. Global strategy on human resources for health: Workforce 2030, Geneva (Switzerland), World Health Organization; 2016. 55 p.

67 Farrell G, Shafiei T, Chan S. Patient and visitor assault on nurses and midwives: an exploratory study of employer 'protective' factors. *Int J Ment Health Nurs* [Internet]. 2014 Feb [cited 2023 Mar 3];**23(1):**88–96. Available from: <https://onlinelibrary.wiley.com/doi/10.1111/inm.12002>. doi: 10.1111/inm.12002

68 Bryant-Genevier J, Rao C, Lopes-Cardozo B, et al. Symptoms of depression, anxiety, post-traumatic stress disorder, and suicidal ideation among state, tribal, local, and territorial public health workers during the COVID-19 pandemic - United States, March-April 2021. *MMWR Morb Mortal Wkly Rep*. 2021 Jul 2 [cited 2023 Feb 22];**70(26):**947–952. Available from: <https://www.cdc.gov/mmwr/volumes/70/wr/mm7026e1.htm?s_cid=mm7026e1_w>. doi: 10.15585/mmwr.mm7026e1. Corrected and republished in: *MMWR Morb Mortal Wkly Rep*. 2021 Dec 03;70(48):1679

69 Centers for Disease Control and Prevention; National Institute for Occupational Safety and Health. Workplace Violence Prevention Strategies and Research Needs: Report from the Conference Partnering in Workplace Violence Prevention: Translating Research to Practice, November 17–19, 2004, Baltimore, Maryland; 2006. 38 p.

70 Wassell J, Workplace violence intervention effectiveness: A systematic literature review. *Safety Science* [Internet]. 2009 Oct [cited 2023 Mar 21];**47(8)**. Available from: <https://www.sciencedirect.com/science/article/pii/S092575350800218X?via%3Dihub>. doi: <https://doi.org/10.1016/j.ssci.2008.12.001>

71 Kumari A, Sarkar S, Ranjan P, et al. Interventions for workplace violence against health-care professionals: a systematic review. *Work* [Internet]. 2022 Oct 17 [cited 2023 Mar 12];**73(2):**415–427. Available from: <https://content.iospress.com/articles/work/wor210046>. doi: 10.3233/WOR-210046

72 Baby M, Gale C, Swain N. A communication skills intervention to minimise patient perpetrated aggression for healthcare support workers in New Zealand: a cluster randomised controlled trial. *Health Soc Care Community* [Internet]. 2018 Sep 2 [cited 2023 Mar 3];**27(1):**170–181. Available from: <https://onlinelibrary.wiley.com/doi/10.1111/hsc.12636>. doi: 10.1111/hsc.12636

73 Larson L, Finley J, Gross T, et al. Using a potentially aggressive/violent patient huddle to improve health care safety. *Jt Comm J Qual Patient Saf* [Internet]. 2019 Jan 10 [cited 2023 Apr 3];**45(2):**74–80. Available from: <https://www.jointcommissionjournal.com/article/S1553-7250(18)30103-X/pdf>. doi: 10.1016/j.jcjq.2018.08.011

74 Hardy S, Bennett L, Rosen P, Carroll S, White P, Palmer-Hill S. The feasibility of using body worn cameras in an inpatient mental health setting. *Mental Health in Family Medicine* [Internet]. 2017 [cited 2023 Mar 7];**13**. Available from: <https://www.researchgate.net/publication/318713534_The_Feasibility_of_Using_Body_Worn_Cameras_in_an_Inpatient_Mental_Health_Setting>. doi: 10.25149/1756-8358.1301001

75 Quigley P, Votruba L, Kaminski J. Impact of patient-engaged video surveillance on nursing workforce safety: patient aggression/violence. *J Nurs Care Qual* [Internet]. 2019 Nov 14 [cited 2023 Feb 23];**35(3):**213–219. Available from: <https://www.ncbi.nlm.nih.gov/pmc/articles/PMC7249483/>. doi: 10.1097/NCQ.0000000000000450

76 Nurses applaud introduction of federal legislation to prevent workplace violence in health care, social service settings. National Nurses United [Internet]. 2022 May 11 [cited 10 Feb 2023]. Available from <https://www.nationalnursesunited.org/press/nurses-applaud-introduction-federal-legislation-to-prevent-workplace-violence-in-health-care>

77 Casteel C, Peek-Asa C, Nocera M, et al. Hospital employee assault rates before and after enactment of the California hospital safety and security act. *Ann Epidemiol* [Internet]. 2009 Feb [cited 2023 Mar 17];**19(2):**125–33. Available from: <https://www.sciencedirect.com/science/article/pii/S1047279708003487?via%3Dihub>. doi: 10.1016/j.annepidem.2008.10.009

78 Odes R, Chapman S, Ackerman S, Harrison R, Hong O. Differences in hospitals' workplace violence incident reporting practices: a mixed methods study. *Policy Polit Nurs Pract* [Internet]*.* 2022 Mar 23 [cited 2023 Mar 18];**23(2):**98–108. Available from: <https://journals.sagepub.com/doi/full/10.1177/15271544221088248>. doi: 10.1177/15271544221088248

79 Occupational Safety and Health Administration. Guidelines for preventing workplace violence for healthcare and social service workers. [Internet]: U.S. Department of Labor: Occupational Safety and Health Administration; 2016 [cited 2023 April 18]. Available from <https://www.osha.gov/sites/default/files/publications/osha3148.pdf>

80 Arnetz J. The Joint Commission’s new and revised workplace violence prevention standards for hospitals: a major step forward toward improved quality and safety. *Jt Comm J Qual Patient Saf* [Internet]. 2022 Feb 5 [cited 2023 Apr 2];**48(4):**241–245. Available from: <https://psnet.ahrq.gov/issue/joint-commissions-new-and-revised-workplace-violence-prevention-standards-hospitals-major>. doi: 10.1016/j.jcjq.2022.02.001

81 Barach P, Small S. Reporting and preventing medical mishaps: lessons from non-medical near miss reporting systems. *BMJ* [Internet]. 2000 Mar 18 [cited 2023 Mar 3];320(7237):759–63. Available from: <https://www.bmj.com/content/320/7237/759>. doi: 10.1136/bmj.320.7237.759
